# Multiple Phosphatidylinositol 3-Kinases Regulate Vaccinia Virus Morphogenesis

**DOI:** 10.1371/journal.pone.0010884

**Published:** 2010-05-28

**Authors:** Shannon McNulty, William Bornmann, Jill Schriewer, Chas Werner, Scott K. Smith, Victoria A. Olson, Inger K. Damon, R. Mark Buller, John Heuser, Daniel Kalman

**Affiliations:** 1 Microbiology and Molecular Genetics Graduate Program, Emory University School of Medicine, Atlanta, Georgia, United States of America; 2 MD Anderson Cancer Center, University of Texas, Houston, Texas, United States of America; 3 Department of Molecular Microbiology and Immunology, Saint Louis University Health Sciences Center, St. Louis, Missouri, United States of America; 4 Poxvirus Team, Poxvirus and Rabies Branch, Division of Viral and Rickettsial Diseases, National Center for Zoonotic, Viral and Enteric Diseases, Centers for Disease Control and Prevention, Atlanta, Georgia, United States of America; 5 Department of Cell Biology, Washington University School of Medicine, St. Louis, Missouri, United States of America; 6 Department of Pathology and Laboratory Medicine, Emory University School of Medicine, Atlanta, Georgia, United States of America; University of California San Francisco, United States of America

## Abstract

Poxvirus morphogenesis is a complex process that involves the successive wrapping of the virus in host cell membranes. We screened by plaque assay a focused library of kinase inhibitors for those that caused a reduction in viral growth and identified several compounds that selectively inhibit phosphatidylinositol 3-kinase (PI3K). Previous studies demonstrated that PI3Ks mediate poxviral entry. Using growth curves and electron microscopy in conjunction with inhibitors, we show that that PI3Ks additionally regulate morphogenesis at two distinct steps: immature to mature virion (IMV) transition, and IMV envelopment to form intracellular enveloped virions (IEV). Cells derived from animals lacking the p85 regulatory subunit of Type I PI3Ks (p85α^−/−^β^−/−^) presented phenotypes similar to those observed with PI3K inhibitors. In addition, VV appear to redundantly use PI3Ks, as PI3K inhibitors further reduce plaque size and number in p85α^−/−^β^−/−^ cells. Together, these data provide evidence for a novel regulatory mechanism for virion morphogenesis involving phosphatidylinositol dynamics and may represent a new therapeutic target to contain poxviruses.

## Introduction

Orthopoxviruses, including vaccinia virus (VV), monkeypox (MPX), and variola (VarV), are large dsDNA viruses that cause characteristic umbilicated vesiculo-pustular skin lesions (“pox”). VarV is the causative agent of smallpox, and VV is used for vaccination against smallpox. Although smallpox has been eradicated, naturally occurring poxviruses are still of concern to humans. Molluscum contagiosum is prevalent in children and immunocompromised individuals [1], [2], while MPX is endemic in Africa and has the potential for dissemination as evidenced by the 2003 outbreak in the United States [3], [4].

Infection is initiated upon entry of either of two forms of the virus. The first, called the Intracellular Mature Virus (“IMV”, and also called Mature Virion “MV”), consists of a brick-shaped viral core surrounded by one or two lipid bilayers derived from an ER-golgi intermediate compartment (ERGIC) [5], [6], [7], [8]. IMV enter the cell either through direct fusion with the plasma membrane or by internalization and fusion with an endocytic compartment [9], [10], [11], [12]. A second infectious form of the virus, called the extracellular enveloped virus (“EEV”, and also called Enveloped Virus “EV”) [13], is released from the cell surface and consists of an IMV enveloped in additional host-cell derived membranes. Entry of EEV requires disruption of the outer viral membrane either at the plasma membrane or in endosomes [14], [15], [16]. After delivery of the viral core to the cytoplasm, early viral gene transcription and translation initiates replication and morphogenesis.

Morphogenesis is regulated at multiple steps [17], which have been characterized by electron microscopy in conjunction with mutant viruses containing deletions or temperature-sensitive alleles of viral proteins. Morphogenesis ensues approximately four hours after entry with the appearance of viral crescents [18], [19], which consist of semi-spherical membranes containing viral proteins apposed to the viroplasm. The crescent membrane extends to encase viroplasm, forming a spherical immature virion (IV). The transition from IV to IMV depends on several events, including proteolysis of core proteins such as A3 (P4b), A10 (P4a), and L4 (P25K), and formation of disulfide bonds within nascent viral proteins L1 and E10 [20], [21], [22], [23]. IMV formation also depends on the viral kinase F10 [24], core proteins P4a, A10, P4b, A3, P25, and L4 [25], and on redox proteins G4 and E10 [26], [27], [28], [29]. Mutations in these proteins result in slightly different phenotypic defects, including an inability to form complete crescents, misincorporation of the viroplasm with or without crescent resolution, malformed cores, or incomplete virion maturation.

Once formed, a subset of IMVs traffic along microtubules to a juxta-nuclear region where they are enveloped in host-cell membranes derived from endosomes or from the Golgi apparatus to form IEV (Intracellular Enveloped Virions) [30], [31], [32], [33], [34]. The mechanisms controlling envelopment are less well understood, but evidence suggests the involvement of F12 and A36, F13, A33, A34, A56 and B5 [13], which physically associate or facilitate localization of other proteins. For example, F13 and A34 have been shown to regulate the cellular trafficking of B5 and its incorporation into the IEV [35], [36]. Ultimately, the interaction of these proteins allows the virus to utilize host membranes for envelopment.

While much information is available about the viral proteins that govern entry and maturation, much less is known about the host cell factors that contribute to trafficking and membrane wrapping processes. In this paper, we provide evidence that maturation of several orthopoxviruses, including VV, MPX and ectromelia, is regulated by host phosphatidylinositol-3 family kinases (PI3K). PI3Ks catalyze addition of a phosphate group to the D3 hydroxyl of the inositol ring of phosphatidylinositol [37], [38], [39], and regulate many cellular processes including growth factor and hormonal signaling, autophagy, nutrient sensing, and endosomal trafficking [37], [40], [41], [42].

PI3Ks are encoded by multiple genes and are categorized into three classes based on domain structure and substrate preference (reviewed by [38]). Kinases within each class are controlled by regulatory proteins. These proteins are encoded by multiply spliced genes, which can generate additional signaling complexity. Genes encoding PI3Ks are frequently mutated in human cancers, making this class of enzymes an important pharmaceutical target [43], [44], [45], [46].

Many viruses, including VV and Cowpox, activate PI3Ks to inhibit cellular apoptosis [47], [48], [49]. Here we demonstrate that poxviruses additionally utilize PI3K at several distinct stages of morphogenesis, including early and late gene expression, late protein trafficking and envelopment, and maturation of virions, suggesting a much broader role for this class of host molecules than has been previously recognized.

## Materials and Methods

### Cells, viruses and plaque assays

BSC40 cells (ATCC) were grown in DMEM or RPMI (Cellgro, MediaTech, Inc; Manassas, VA) supplemented with 10%FBS (Atlanta Biologicals; Norcross, GA) and 10 IU/mL Penicillin and 10 µg/mL streptomycin (P/S; Cellgro, MediaTech, Inc.; Manassas, VA). BHK cells were grown in alpha-MEM (Gibco; Carlsbad, CA) supplemented with 10%FBS and P/S; p85WT and p85α^−/−^ cells were grown in DMEM with 10%FBS and P/S, while p85α^−/−^β^−/−^ cells were grown in DMEM with 15%FBS and P/S. All cells were grown at 37°C in a 5% CO_2_ incubator. p85-deficient cells were provided by Lewis Cantley. Mouse embryonic fibroblasts were isolated from embryos that were heterozygous or homozygous for knockout of the PI3K-family related genes PIK3R1 and PIK3R2 [50], [51]. Viral strains were grown and propagated as previously described [52]. All strains were titered on BSC40 cells.

### PI3K Inhibitors

AS605240 and AS604850 were chemically synthesized as described [53] and are referred to in the text as AS1 and AS2, respectively. The PI3K-alpha Inhibitor 2 #B0304 or 3-(4-Morpholinothieno[3,2-d]pyrimidin-2-yl)phenol, referred to as B0304, was obtained from Echelon Biosciences, and LY294002 was obtained from Sigma-Aldrich (St. Louis, MO).

### Plaque assays and calculation of IC_50_ Values

To calculate IC_50_ values, PI3K inhibitors were added post-viral adsorption to poxviral-infected monolayers. Experiments with vaccinia virus were conducted at Emory University under BSL-2 conditions. 100PFU of vaccinia virus strain WR was diluted in 500 µL of 2% FBS/DMEM and added to monolayers of naïve BSC40, p85WT, p85α^−/−^, or p85α^−/−^β^−/−^ cells in 12 well dishes. Virus was allowed to adsorb to and enter the cells for one hour at 37°C in 5% CO_2_. After 1 hour, virus was removed and monolayers were washed twice with 1 mL PBS. Media was then replaced with 10% FBS/DMEM containing PI3K inhibitors at different concentrations. Drugs were resuspended in 100% DMSO, and DMSO controls were performed in conjunction with assays using PI3K inhibitors. Two days after infection, monolayers were fixed and stained with crystal violet solution (0.1% crystal violet and 20% ethanol). The concentration of drug needed to reduce plaque numbers by half (IC_50_) was calculated by fitting the data to a linear regression model using Prism software (GraphPad Prism Software, Inc., La Jolla, CA). Experiments with ectromelia virus were performed at the Saint Louis University School of Medicine. For these experiments, ECTV-Moscow strain p4 was used to infect BSC-1 cells at 50–75 plaques/well of a 12-well culture plate for one hour in DMEM (Lonza, Basel, Switerland) supplemented with 2% Fetal Clone II (FCII, Hyclone, Thermo Scientific, Pittsburgh, PA). Serial dilutions of the compounds were made in DMEM-2% FCII and added to the infected cells along with DMEM-5% FCII-1% carboxymethylcellulose (Sigma-Aldrich; St. Louis, MO). The cultures were incubated 4–5 days and stained with crystal violet (0.13% crystal violet, 5% ethanol, 10% formaldehyde) to visualize plaque formation. IC_50_ values were calculated as described. Experiments with monkeypox were performed at the Centers for Disease Control and Prevention (CDC) in Atlanta, GA under BSL2+ conditions. For these experiments, 50PFU/mL of strain V79-1-005 in 2% FBS RPMI (Gibco; Carlsbad, CA) was added to BSC40 cells for one hour at 35.5°C in a 6% CO_2_ incubator. After one hour virus was removed and monolayers were washed twice with 2% RPMI media. Media was replaced with 2%RPMI/1%DMSO containing PI3K inhibitors at different concentrations and cells were incubated at 35.5°C in 6% CO_2_ for three days. After three days, monolayers were fixed and stained with 2X CV. IC_50_ and IC_20_ values were calculated as described.

### Western analysis

BHK or p85 cells were grown in 10 cm tissue culture dishes and infected at an MOI of five with vaccinia virus strain Western Reserve (WR). Briefly, virus was incubated in a minimal amount of 2% FBS/DMEM for one hour at 37°C to allow virus to adsorb and enter monolayers. Monolayers were washed once with PBS and media was replaced with 10%FBS DMEM with or without PI3K inhibitors for an additional sixteen hours. Western analysis of P4b/4b was done in conjunction with infection periods of 6, 16 or 24 hours. Cells were lysed in RIPA buffer (Cell Signaling; Beverly, MA). The amount of protein was quantified using the Bio-Rad Dc Protein Assay Kit (Bio-Rad; Hercules, CA), and 50 µg was separated by SDS-PAGE. For acid-bypass experiments p85WT and p85-deficient cells were grown in 10 cm tissue culture dishes and infected and analyzed by the same protocol as Mercer and Townsley *et al.* [54], [55]. Briefly, WR was added to cells at an MOI of 5 in 2 mL of pre-cooled Opti-Pro SFM supplemented with P/S and 20 mL 200 µM L-Glutamine (Gibco; Carlsbad, CA) and virus was allowed to bind to cells at 4°C for one hour. Cells were treated with pre-warmed PBS and 1 mM MES, pH of 5 for five minutes at 37°C in 5% CO_2_. Media was then removed and the monolayers washed once with 5 mL pre-warmed 10% FBS/DMEM, and media was replaced with 10% FBS/DMEM. After 2 hours, the infection was stopped by rinsing the cells with PBS and lysing the cells with RIPA Buffer (Cell Signaling; Beverly, MA). Proteins were quantified as above. To generate blots, proteins were then transferred to nitrocellulose, and the membranes were blocked with 3% BSA in Tris-buffered saline containing 0.5% Tween-20 (TBST) for one hour. Membranes were probed with antibodies in the blocking solution for an additional hour. For infections with B5-GFP [56] and F13-GFP VV [57], membranes were probed with GFP antibody (1∶1000, Living Colors Full-length A.v. Polyclonal Antibody, Clonetech; Mountain View, CA). Anti-L1 (10F5) was a gift from Jay Hooper at USAMRIID and was used at 1∶1000 dilution for western blots and 1∶50 for microscopy. Anti-P4b/4b was a gift from Bernard Moss at NIH, and was used at 1∶3500. Anti-E3 was a gift from Stuart Isaacs at University of Pennsylvania and was used at 1∶1000. Bands were detected using anti-Mouse or anti-Rabbit HRP conjugated antibodies (GE Healthcare; UK) and blots were developed using Pierce ECL Western Blotting Substrate (Thermo Scientific; Waltham, MA). Independent experiments were performed in duplicate to confirm Western blot results. Fold change was calculated from a representative experiment by quantifying band intensity using Adobe Photoshop v10.0.1 and samples were normalized against Tubulin controls. Data is expressed as the fold change relative to untreated samples.

### Microscopy

Deconvolution microscopy was carried out as previously described [52]. Spinning disk microscopy was performed at the Heuser lab on a Zeiss Axioplan 2 with Yokagawa CSU 10 confocal scanner unit.

### Growth Curves

BHK or p85WT, p85α^−/−^ or p85α^−/−^β^−/−^ cells were grown to ∼80% confluence in triplicate 12-well tissue culture dishes. Prior to infection cell numbers were quantified, and cells were infected at an MOI of 5, 1, 0.01, 0.001, or 0.0001 in 2%FBS/DMEM for one hour at 37°C with vaccinia virus strain WR. After adsorption, monolayers were washed twice with 1 mL PBS and media was replaced with 10%FBS DMEM with or without PI3K inhibitors. AS1 and AS2 were used at 50 µM, Rifampicin at 0.1 mg/mL, and the carrier DMSO was maintained at 0.5%. At each time point, the supernatant was removed and the monolayers were scraped into 0.5 mL 2%FBS/DMEM. Cell-associated virus was isolated by freezing and thawing cells three times, and then centrifuging at 500xg for three minutes to remove cell debris. Supernatants containing released virus or cell-associated virus were diluted in 2%FBS/DMEM and then added to naïve (uninfected) BSC40 monolayers.

### Comet Assays

BSC40 cells were allowed to form confluent monolayers in 6-well dishes. Media was removed and replaced with 100PFU/well IHD-J vaccinia virus diluted in 500 µL 2% FBS/DMEM. Virus adsorbed to the cells for one hour at 37°C. After adsorption virus was removed and monolayers were washed twice with 1 mL PBS, and media was replaced with 10%FBS DMEM with or without PI3K inhibitors. AS1 and AS2 were added at 50 µM, while B0304 and LY294002 were added at 20 µM final concentrations. DMSO was added at a final concentration of 0.5%. Infections were stopped after 48 hours with the addition of crystal violet stain.

### MTS Assay

Serial dilutions of PI3K compounds were made in DMEM (Lonza; Basel, Switerland) supplemented with 2% Fetal Clone II (Hyclone, Thermo Scientific, Pittsburgh, PA). Compound dilutions were mixed with BSC-1 cells at 5000 cells per reaction in 100 µl total volume. At the specified time point, 20 µl of MTS [3-(4,5-dimethylthiazol-2-yl)-5-(3-carboxymethoxyphenyl)-2-(4-sulfophenyl)-2H-tetrazolium] solution (CellTiter 96 Aqueous One Solution Cell Proliferation Assay, Promega; Madison, WI) was added to each reaction and incubated 2–4 hours. The absorbance was measured at 490 nm and used to determine percent cell viability for each reaction compared to untreated cells. Compound concentration was plotted versus percent cell viability to determine the concentration at which the cell viability was reduced by 50% (CC_50_).

### Trypan Blue Exclusion Assay

BHK cells were grown to ∼80% confluency in 12-well tissue culture dishes and treated with 50 µM AS1 or AS2 or treated with 0.5% DMSO in 10%FBS/DMEM. At each time point, cells were trypsinized with 0.5 mL 0.25% Trypsin/EDTA (Cellgro, MediaTech, Inc.; Manassas, VA), and combined with removed supernatant. Cells were spun down at 1500xg for five minutes. A supernatant volume of 800 µl was removed and replaced with 0.4% (w/v) Trypan Blue in saline (Cellgro, MediaTech, Inc.; Manassas, VA) and cells were resuspended. Viable (clear) and nonviable (blue) cells were counted on a hemacytometer. Samples were scored from duplicate tissue culture wells.

### TUNEL Assay

BHK or p85WT, p85α^−/−^ or p85α^−/−^β^−/−^ cells were grown to near confluency on slides prepared for microscopy. Cells were treated with 50 µM AS1 or AS2, and 20 µM LY294002 or B0304 in 10%FBS/DMEM for sixteen hours. Apoptosis was detected using the ApoTag Fluorescein *in situ* Apoptosis Detection Kit (Chemicon International, Millipore; Billerica, MA). As a positive control cells were treated with 20U DNase1 (Epicentre; Madison, WI) for 10 minutes at room temperature post-fixation. Each experiment was carried out with duplicate microscope slides. Two images were taken per slide, and ∼700 cells were counted per condition.

### Electron Microscopy

Electron microscopy was performed by the Heuser lab at Washington University at St. Louis. Briefly, BHK, HeLa or p85WT or p85α^−/−^β^−/−^ cells were grown on Thermanox dishes (Nunc, Fisher Scientific), and infected with MVA or B5-GFP WR virus as previously described [58], [59]. After a 17-hour infection period cells were fixed and processed for microscopy. For glutaraldehye/KMnO_4_ fixation and staining, cultures were fixed for 1 hour at room temperature with 2% glutaraldehyde in NaHCa Buffer (30 mM HEPES, pH 7.4 with NaOH, 100 mM NaCl, 2 mM CaCl, and 10 mM MgCl_2_), washed 2 times with NaHCa buffer for 5 minutes, washed for 15 minutes with quenching solution (50 mM glycine, 50 mM lysine, 50 mM NH_4_Cl in NaHCa Buffer), followed by 5× washes with NaHCa buffer (5 minutes each wash) and 2 washes of 0.1M NaCl (10 minutes each wash). Samples were stained with 0.5% KMnO_4_ in 0.1M NaCl, washed twice with 0.1M NaCl and dehydrated with 25%, 50%, 75%, 95% and 100% ethanol over 20 minutes. Samples were embedded using the Protocol for Fast Embedding. Coverslips were dipped sequentially in: 100% ethanol, 100% ethanol, 100% propylene oxide, 100% propylene oxide, 50/50 (v/v) propylene oxide and epoxy resin, 50/50 (v/v) propylene oxide and epoxy resin, 100% epoxy resin, 100% epoxy resin, inverted over Beem capsules and allow to polymerize at 65°C. For glutaraldehyde/OsO_4_ fixation and staining cultures were rinsed in Ringer's solution and fixed for 30–60 minutes at room temperature in 2% glutaraldehyde in NaHCa buffer. Post-fixation cells were rinsed twice in NaHCa buffer and stained with TA (1% tannic acid, 0.075% saponin in NaHCa Buffer) for 15 minutes at room temperature. Cells were then rinsed twice with NaHCA buffer, twice with 0.1M cacodylate buffer, pH 7.4, and postfixed with 0.5% OsO_4_ in 0.1 M cacodylate buffer, pH 7.4 for 15 minutes at room temperature. After OsO_4_ samples were rinsed twice in 0.1 M cacodylate buffer pH 7.4 (5 minutes each wash) and twice in 0.1 M sodium acetate buffer, pH 5.2 (20 minutes each wash). Samples were then stained with 4% uranyl acetate in 50 mM sodium acetate buffer for 15 minutes in the dark at room temperature. Samples were then rinsed twice in 0.1 M sodium acetate buffer (5 minutes each rinse) and dehydrated as above. Samples were embedded in resin as described above. Samples were prepped for EM as previously described [60].

## Results

### PI3Ks regulate plaque morphology

**Figure 1:**
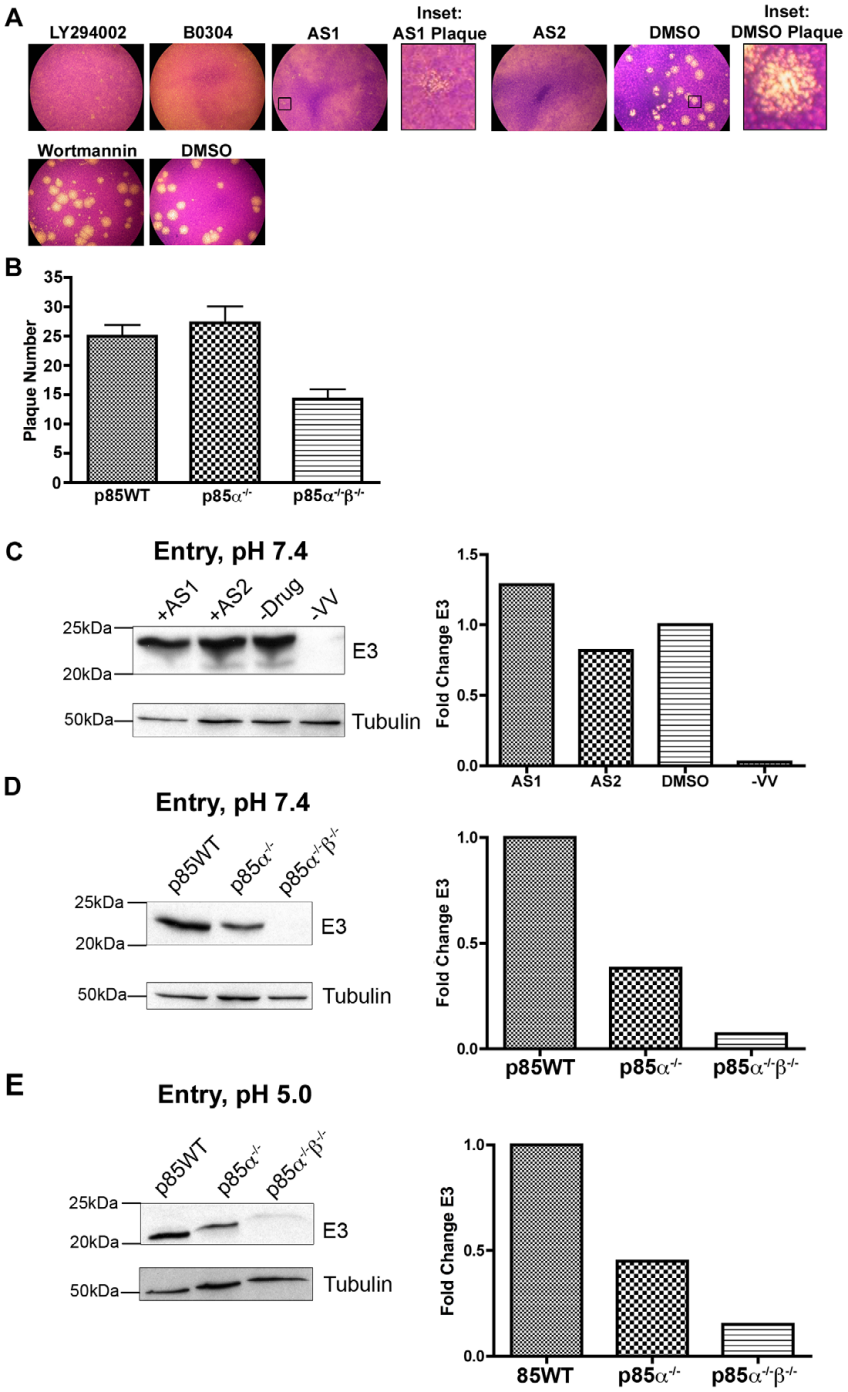
PI3Ks regulate plaque morphology and early protein production. **A.** PI3K inhibitors reduce plaque size in BSC40 cells. Cells were infected with 100 PFU of vaccinia virus (VV), strain WR. Inhibitors were added one hour post viral entry. First row: LY294002, 20 µM; B0304, 20 µM; AS1, 50 µM; AS2, 50 µM (LY294002 and B0304 are in 0.2% DMSO and AS1 and AS2 are in 0.5% DMSO); 0.5% DMSO alone. Insets show representative plaques from 50 µM AS1 and 0.5% DMSO treated wells. Second row: 10 µM Wortmannin in 0.1% DMSO did not appear to reduce VV plaque size; 0.1% DMSO control. Results are from three independent trials. **B.** p85WT and p85α^−/−^ cells form two times more plaques than p85α^−/−^β^−/−^ cells. Cells were infected with equal amounts of virus and monolayers were stained with crystal violet 48 hours post infection. Results are from eight independent trials. **C.** PI3K inhibitors do not decrease early viral protein expression. BSC40 cells were infected at pH 7.4 and MOI = 5 with VV and PI3K inhibitors were added 1 hour post-viral entry and allowed to infect for an additional 2 hours. Proteins were subjected to western analysis by anti-E3 mAb and anti-Tubulin mAb. For quantification (right) bands were normalized to tubulin loading control and data is expressed as the fold change relative to DMSO-treated samples. **D.** Early protein expression is reduced in p85-deficient cells. Cells were infected at an MOI = 5 with VV at pH = 7.4 and analyzed as in C. **E.** Early protein expression is reduced in p85-deficient cells. Cells were infected at an MOI = 5 with VV. Virus was bound to cells at 4°C, after which cells were moved to 37°C with PBS, pH = 5. Following a 2 hour infection, equal amounts of protein were subjected to western analysis with anti-E3 mAb or anti-Tubulin, and quantitated as in C.

To identify host kinases utilized by VV, we screened a small directed library, which included approximately 350 novel and previously described inhibitors, for those that decreased the size of plaques. Monolayers were treated with compounds at a final concentration of 50–100 µΜ. The construction and composition of the library is described elsewhere (S.M, D.K., and W.G.B. in preparation), but consisted of compounds that inhibited tyrosine kinases as well as other kinases. Here we focus on two inhibitors present in the library called AS1 (AS605240) and AS2 (AS604850), which decreased plaque size to pinpoints (Figure 1A).

AS1 and AS2 inhibit the p110-gamma subunit of the type 1 PI3K; but they also inhibit the other PI3K subtypes at higher concentrations (Table 1) [61]. To confirm that inhibition of PI3Ks by AS1 and AS2 could account for the pinpoint plaques, several well-characterized PI3K inhibitors were tested including wortmannin and LY294002, and a p110-alpha specific inhibitor B0304. Addition of LY294002 and B0304 after adsorption of the virus decreased plaque size relative to control-treated monolayers (Figure 1A). Wortmannin did not decrease plaque size up to 48 hours after infection, the longest time tested. However, this drug, in contrast to AS1, AS2 B0304 and LY294002, is a covalent inhibitor that reacts with tissue culture components and was not replenished during the experiment [61], [62], [63], [64], [65]. IC_50_ values for AS1, AS2, B0304, and LY294002 were similar for several orthopoxviral species, including VV, MPX, and ectromelia (Table 1). Lighter crystal violet staining was apparent for MPX-infected monolayers. Therefore, IC_20_ values for orthopoxviral infected cells are presented in Table S1. Notably, fold differences in IC_50_ and IC_20_ values were similar for the different orthopoxviruses.

**Table 1:** 50% Inhibitory Concentration (IC_50_) of PI3K inhibitors added post-adsorption to poxvirus-infected monolayers.

	Inhibits:	Vaccinia IC_50_	Ectromelia IC_50_	Monkeypox IC_50_	Other Targets:
**AS1**	p110-gamma	37.4±1.1 µM	13.0±1.3 µM	12.1±3.9 µM	p110-alpha, p110-beta, p110-delta, PKCbII^1^
**AS2**	p110-gamma	44.6±2.1 µM	44.7±14.2 µM	9.3±4.3 µM	p110-alpha, p110-beta, p110-delta^1^
**B0304**	p110-alpha	9.8±0.3 µM	1.8±0 µM	6.1±4.8 µM	p110-beta, p110-delta, p110gamma^2^
**LY294002**	Broad-Spectrum PI3K Inhibitor^3^	44.1±6.9 µM	14.4±4.2 µM	20.3±10.7 µM	

1 [61]

2 [108]

3 [65]

To ensure that PI3K inhibitors were targeting a host and not a viral kinase, we next assessed VV infection in fibroblast cells derived from wild type animals or from animals with homozygous deletions in p85α (85α^−/−^) or in both p85α and p85β (p85α^−/−^β^−/−^), the regulatory subunits of the Type 1 PI3Ks. Because the stability of the catalytic subunit depends on the regulatory subunit, p85α^−/−^ and p85α^−/−^β^−/−^ cells are deficient in type 1 PI3K activity [50], [66]. In accordance with results with PI3K inhibitors, the number of plaques appeared two-fold lower in the p85α^−/−^β^−/−^ cells compared to wild type cells (Figure 1B).

### PI3K Regulation of early protein production

The PI3K inhibitor LY294002 can disrupt eIF4 complexes during VV infection, and reduces both late proteins and virion production [67]. We next determined the stage(s) viral morphogenesis is disrupted in cells treated with the PI3K inhibitors AS1 and AS2 (one hour after adsorption), or in p85-deficient cells. No significant difference in E3 levels, a measure of early protein synthesis, was evident in cells treated with AS1 or AS2 compared to untreated cells (Figure 1C). These data suggest that PI3Ks sensitive to AS1 or AS2 are not required for expression of early viral proteins, but exert their effects at later stages of maturation. In contrast, E3 levels were reduced by 62% and 93%, respectively, in the p85α^−/−^ and p85α^−/−^β^−/−^ cells relative to the p85WT cells (Figure 1D). To confirm that this difference was a result of reduced protein levels, and not due to a defect in endocytic uptake in p85-deficient cells, we adsorbed virus to cells at 4°C and reduced the pH to catalyze entry [55]. Under these conditions, E3 levels were still reduced by 55% and 85%, respectively, in the p85α^−/−^ and p85α^−/−^β^−/−^ cells relative to the p85WT cells (Figure 1E), suggesting that the observed reduction in E3 levels in p85-deficient cells (Figure 1D) resulted from a defect in early protein accumulation and not entry. We cannot rule out the possibility that an early p85-dependent transport or transcription step occurs before the addition of inhibitors. This may account for the apparent differences in E3 levels between p85-deficient cells and the inhibitors.

### PI3Ks mediate late protein production

**Figure 2:**
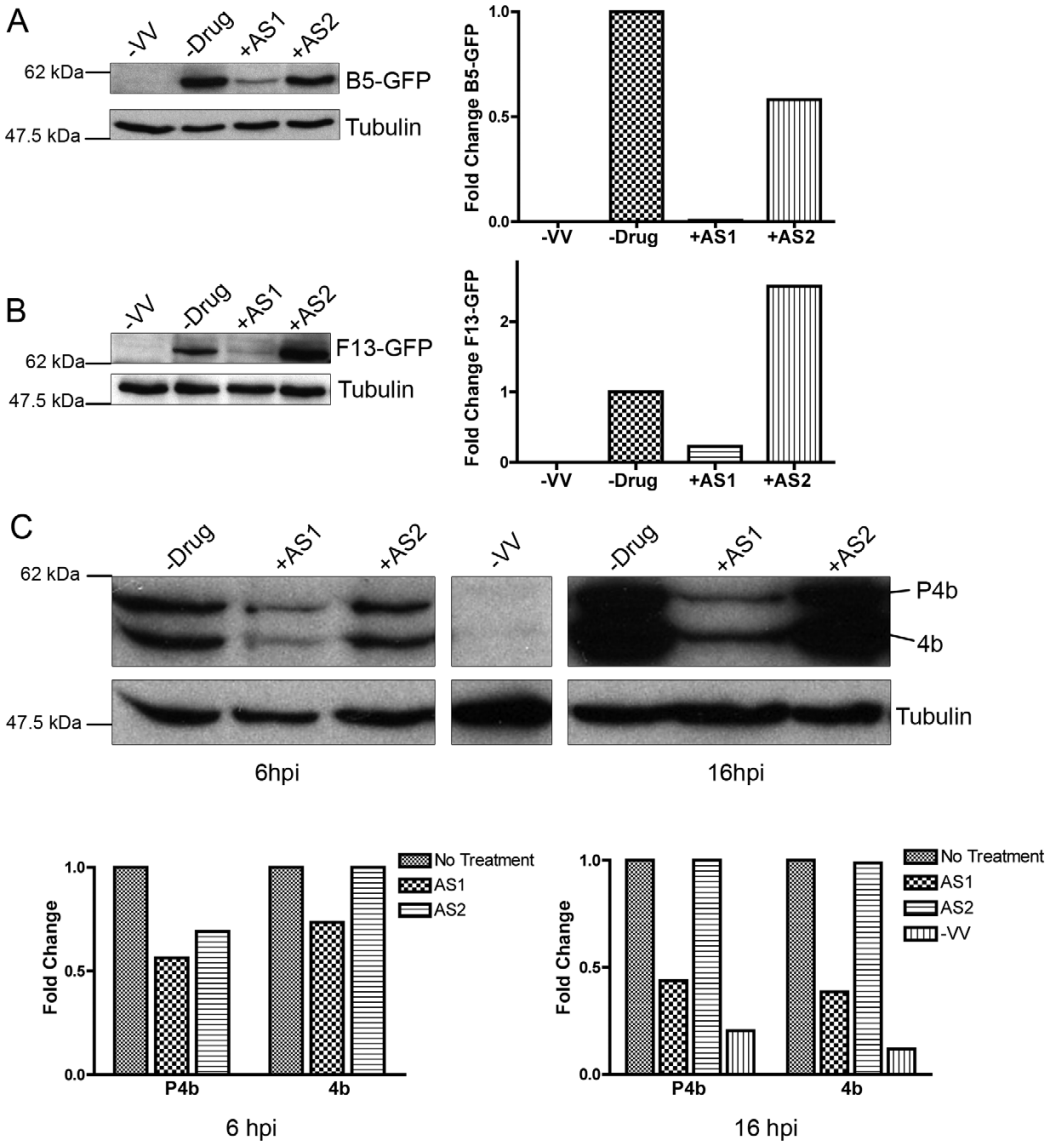
PI3K inhibitors reduce late viral protein expression. BHK cells were infected with VV at an MOI of 5, and PI3K inhibitors were added following entry. After 16 hours proteins were subjected to western analysis with various antibodies, including anti-Tubulin to ensure equal loading. Quantitation (right) is described in Methods. Data are expressed as the fold change relative to DMSO-treated samples. **A.** Cells were infected with VV strain WR expressing B5-GFP under its own promoter, and subjected to western analysis with GFP (top) or tubulin (bottom) antibodies. **B.** Cells were infected with VV strain WR expressing F13-GFP, and analyzed as in A. **C**. Cells were infected with VV strain WR for 6 or 16 hours. Filters were probed with P4b/4b or tubulin antibodies. Results are from two or three independent trials.

We next investigated the role of PI3Ks in the production of IEV/EEV proteins using VV strains that express the B5 and F13 as GFP fusions under their own promoters (B5-GFP and F13-GFP). As shown in Figures 2A and 2B, treatment of infected cells post-adsorption with AS1 decreased F13 and B5 accumulation as measured by western analysis with GFP antibody. AS1 also reduced the levels of IMV-specific protein P4b/4b by 43.6% and 30.9% after 6 hours, and by 56% and 61.4% after 16 hours (Figure 2C). Notably, AS1 did not appear to affect the cleavage of the core protein P4b into 4b, a proteolytic step mediated by I7 during viral maturation [68]. In contrast, AS2 did not appear to cause significant decreases in F13, B5 or P4b levels, and did not affect proteolysis of P4b into 4b (Figure 2C). These results demonstrate that AS1 and AS2 appear to inhibit VV maturation through different mechanisms: AS1 inhibits late protein production, whereas AS2 does not.

**Figure 3:**
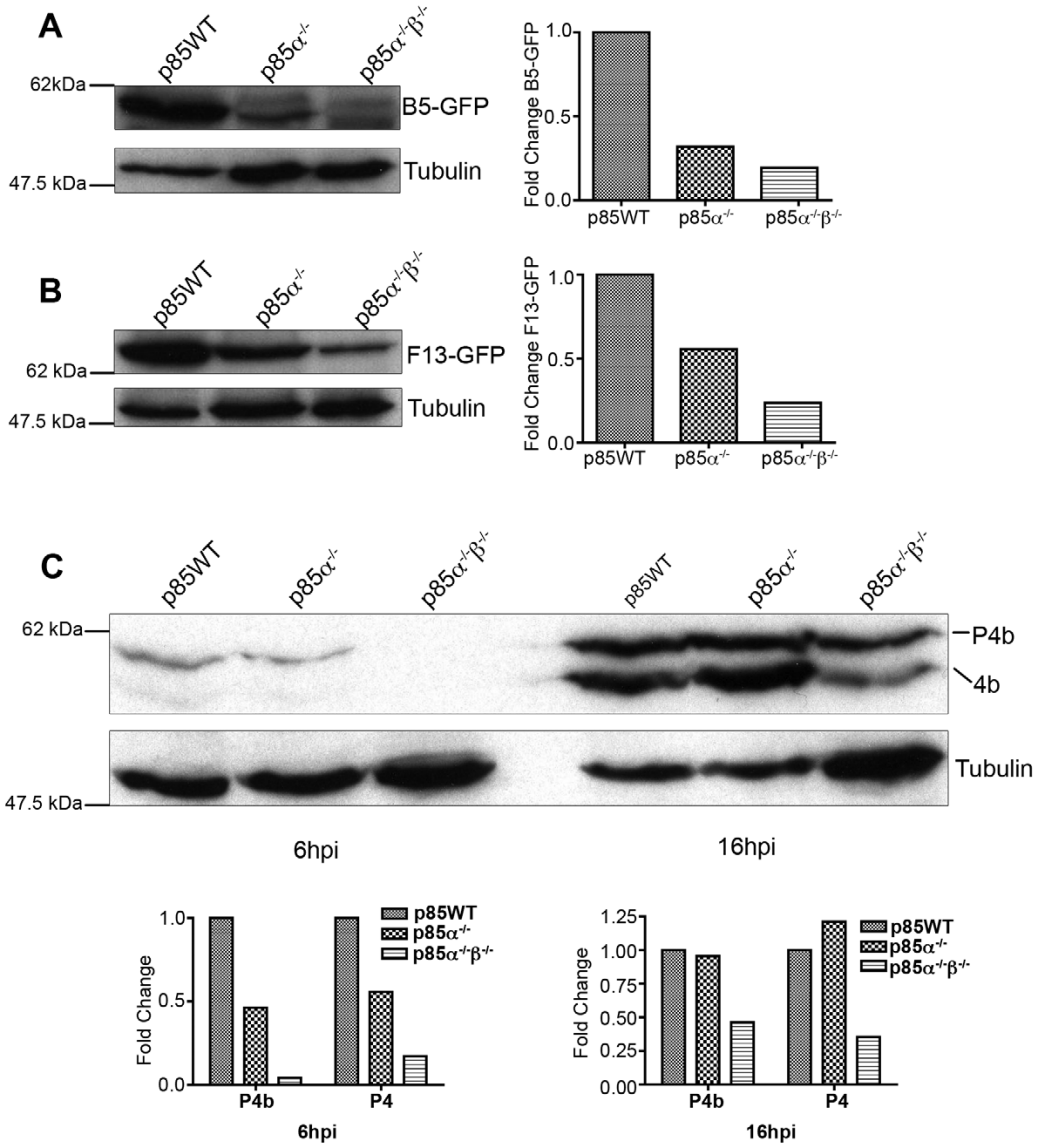
Late viral protein synthesis is reduced in p85-deficient cells. p85-deficient cells were infected with VV at an MOI of 5 for 16 hours and subjected to western analysis with various antibodies. **A.** Cells were infected with VV strain WR expressing B5-GFP under its own promoter. Filters were probed with GFP (top) or tubulin (bottom) antibodies. **B.** Cells were infected with VV strain WR expressing F13-GFP. Filters were probed with GFP (top) or tubulin antibodies. **C**. Cells were infected with VV strain WR for 6 or 16 hours. Filters were probed with P4b/4b or tubulin antibodies. Results are from two or three independent trials.

In accordance with the results with AS1, p85α^−/−^ and p85α^−/−^β^−/−^ cells had reduced levels of F13, B5, and p4b and 4b (Figure 3A, 3B, 3C). In addition, the proteolysis of p4b into 4b does not appear to be significantly inhibited in the p85α^−/−^ and p85α^−/−^β^−/−^ cells (Figure 3C). Notably, the degree of reduction in late proteins appeared correlated with the genetic deficiency of the cell type used; thus, the p85α^−/−^β^−/−^ cells express less late protein than the p85α^−/−^ cells, which express less than control cells. As a control, neither the p85-deficient cells nor the inhibitor-treated cells showed increased TUNEL staining, suggesting that the reduction in late protein levels did not result from increased apoptosis (Figures S1A and S1B). Collectively, these data suggest that PI3Ks may regulate expression of late proteins in several ways: data with p85-deficient cells suggests that prior effects on early protein expression may contribute, whereas data with AS1 suggests that PI3Ks may affect a subsequent step(s). We cannot rule out the possibility that DNA replication may also be regulated by PI3Ks.

### PI3Ks regulate localization of L1, F13 and B5

Previous studies demonstrate that F13 and B5 are required for envelopment of IMV [69], [70], [71], [72], [73], and that F13 regulates B5 movement from the Golgi to post-Golgi vesicles [35]. Because phosphoinositides are important regulators of endosome trafficking and Golgi structure, we hypothesized that PI3K inhibition or absence could disrupt IMV envelopment and, consequently, localization of B5 and F13. To test this, we assessed whether treatment with PI3K inhibitors altered the subcellular localization of F13, B5, and L1 in cells infected with VV F13-GFP, VV B5-GFP or WR.

**Figure 4:**
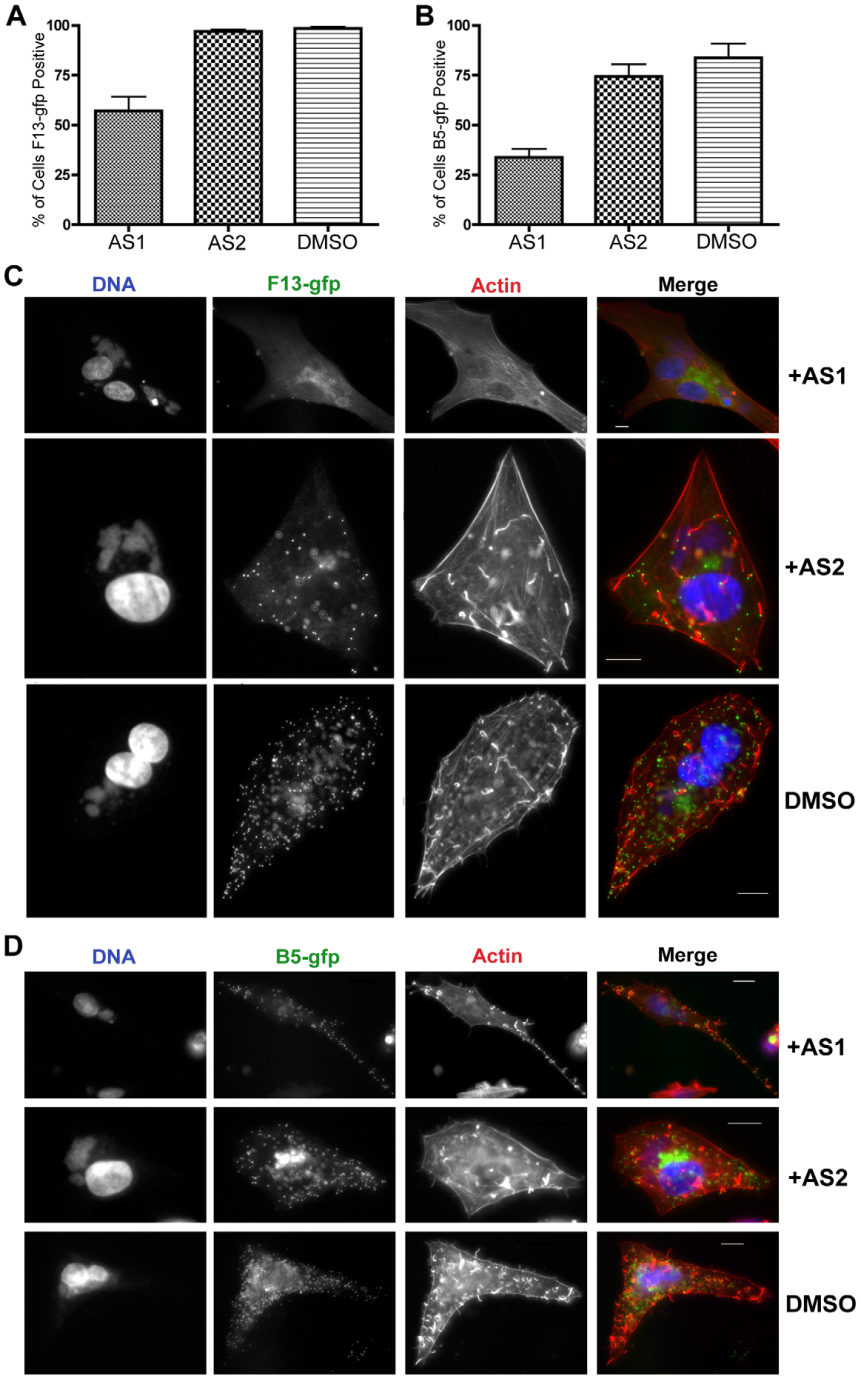
PI3K inhibitors alter the distribution of F13 and B5 in infected cells. **A**. BHK cells were infected with VV F13-GFP, and treated with AS1 (50 µM) or AS2 (50 µM) post-adsoption for 16 hours. Cells were fixed and stained with phalloidin to visualize actin (red) and DAPI to visualize DNA (blue). Approximately 600 cells were counted per condition and cells were scored positive if a FITC (GFP) signal could be detected. **B.** BHK cells were infected with VV B5-GFP, and treated with PI3K inhibitors post adsoption for 16 hours, and stained as in A. Approximately 400 cells were counted per condition. **C.** PI3K inhibitors alter the distribution of F13-GFP in infected cells. Cells were infected and stained as in A. Note that AS1 and AS2 cause a reduction of punctate FITC fluorescence at the cell periphery, which represents virions, as well as a reduction in the number of actin tails. AS1 also caused F13-GFP to localize to the cytoplasm instead of on punctate virions. **D.** PI3K inhibitors alter the distribution of B5-GFP in infected cells. Cells were infected and stained as in B. Note that AS1 and AS2 had similar effects on localization of B5 and F13, namely a reduction in peripheral punctate virions and reduced numbers of actin tails. Results are from three independent trials.

**Figure 5:**
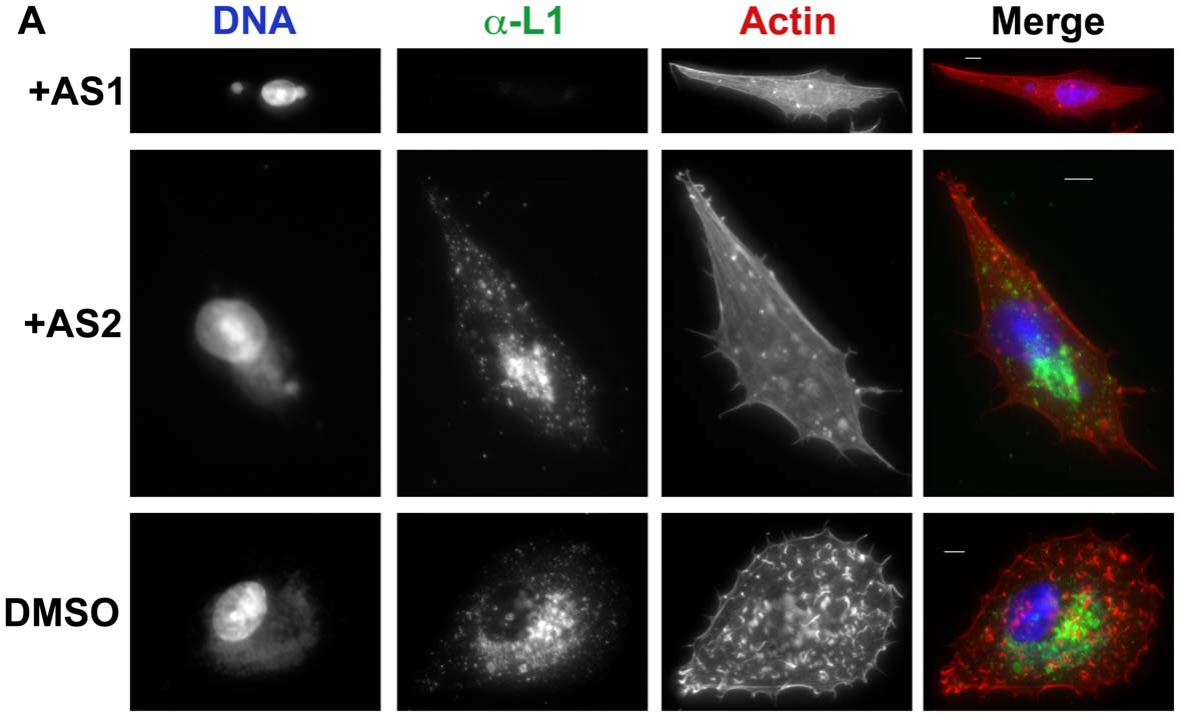
PI3K inhibitors alter the levels of L1 in infected cells. **A**. BHK cells were infected with VV WR, and treated with AS1 (50 µM) or AS2 (50 µM) post-adsoption for 16 hours. Cells were fixed and stained with phalloidin to visualize actin (red), DAPI to visualize DNA (blue), and stained with L1 antibodies to visualize IMV. Note that AS1 causes a reduction of punctate FITC fluorescence in the cells, as well as a reduction in the number of actin tails. AS2 also causes a modest reduction in the number of actin tails relative to DMSO controls. Results are from two independent trials.

AS1 reduced the percentage of infected cells expressing undetectable levels of F13-GFP by ∼50% or B5-GFP by ∼65% (Figure 4A and 4B). Similar effects were seen with the p110α specific inhibitor B0304. In contrast, AS2 had no effect on the percentage of F13-GFP and B5-GFP positive cells (Figure 4A and 4B). In addition, AS1 or B0304 altered the subcellular localization of F13-GFP or B5-GFP fluorescence in individual cells (not shown and Figures 4C and 4D). Control cells typically exhibited multiple actin tails and numerous F13-GFP or B5-GFP positive virions in the cell periphery or on the tips of actin tails. AS1 or B0304 caused a reduction in the number of GFP-positive virions at the cell periphery, and a concomitant reduction in the number of actin tails (e.g. Figures 4C and 4D). Quantitation of actin tails from a representative experiment with AS1 on BSC40 cells is presented in Figure S2. Qualitatively, AS1 caused F13-GFP fluorescence to remain in a diffuse perinuclear compartment rather than puncta (Figure 4C), whereas B5-GFP fluorescence remained punctate (Figure 4D). AS2 was less effective than AS1, but also appeared to reduce both the number of F13-GFP and B5-GFP puncta at the cell periphery, and the number of actin tails relative to DMSO-treated cells (Figures 4C, 4D and S2). Compared to control or AS2-treated cells, the amount of the IMV protein L1 produced in AS1-treated cells was below the level of detection (Figure 5A).

We also performed spinning disk microscopy on HeLa cells treated with PI3K inhibitors and infected with B5-GFP to more clearly resolve membrane localization of late proteins. As seen in z-stack videos, B5-GFP fluorescence in untreated HeLa cells localized to both the plasma membrane and to punctate structures on the plasma membrane (Movie S1). Treatment with AS1 caused B5-GFP to localize to a perinuclear location, albeit with some fluorescence evident in punctate structures (Movie S2). Treatment with AS2 had a similar effect and caused B5 to localize to vacuoles and a small amount on the plasma membrane (Movie S3).

**Figure 6:**
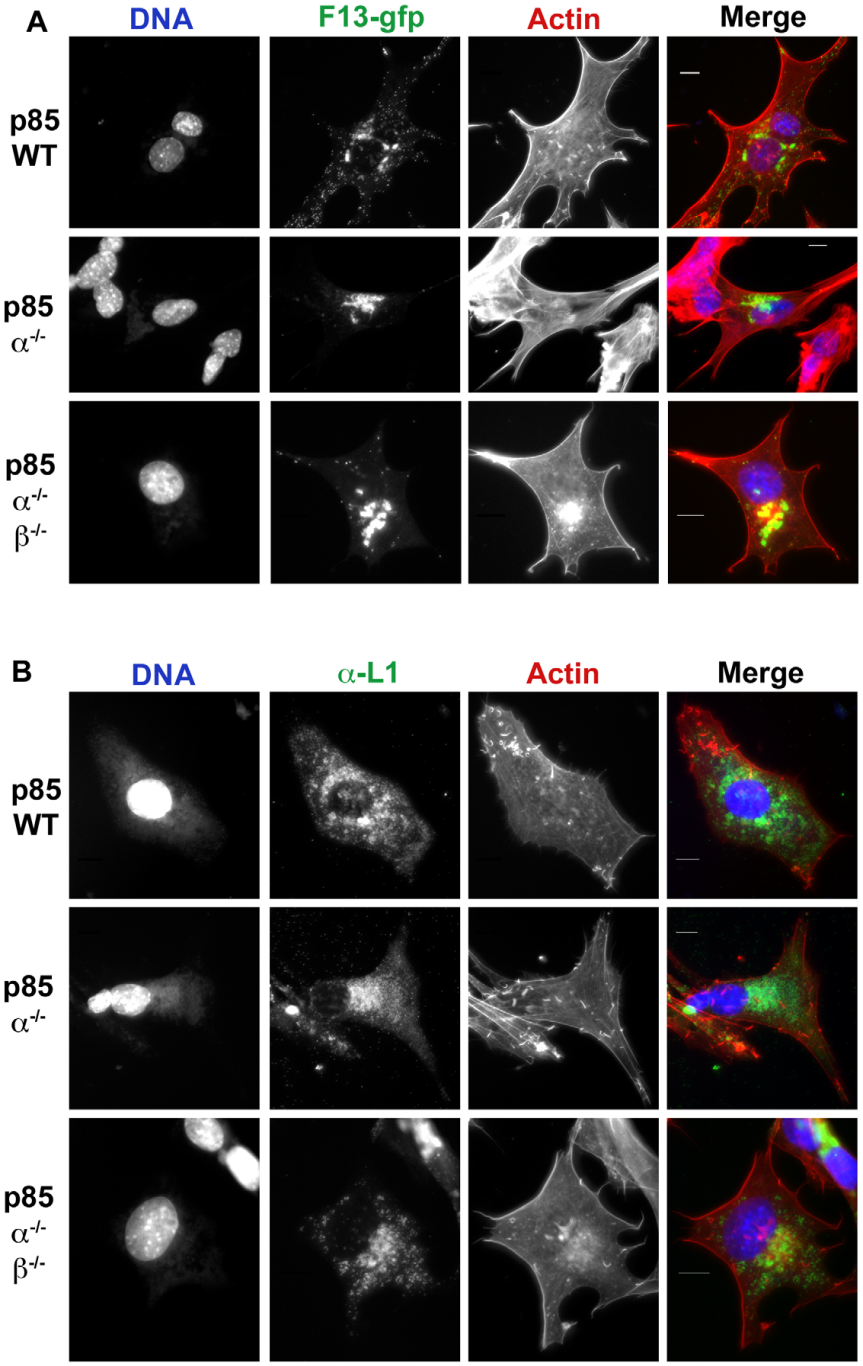
Localization of F13-GFP and L1 in p85-deficient cells. **A.** p85-deficient cells were infected with VV F13-GFP for 16 hours. Cells were fixed and stained with phalloidin to visualize actin (red) and DAPI to visualize DNA (blue). Note that in the p85α^−/−^ and p85α^−/−^β^−/−^ cells there is a reduction of punctate FITC fluorescence at the cell periphery, which represents virions, as well as a reduction in the number of actin tails. F13 remains in a peri-nuclear location in p85-deficient cells. **B.** L1 production and localization in p85-deficient cells. p85-deficient cells were infected with VV, strain WR for 16 hours. Cells were fixed and stained with phalloidin to visualize actin (red), DAPI to visualize DNA (blue), and L1 antibody, followed by FITC conjugated anti-Mouse antibody. No difference in L1 localization the p85-deficient cells compared to wild type cells was apparent. Results are from three independent trials.

**Figure 7:**
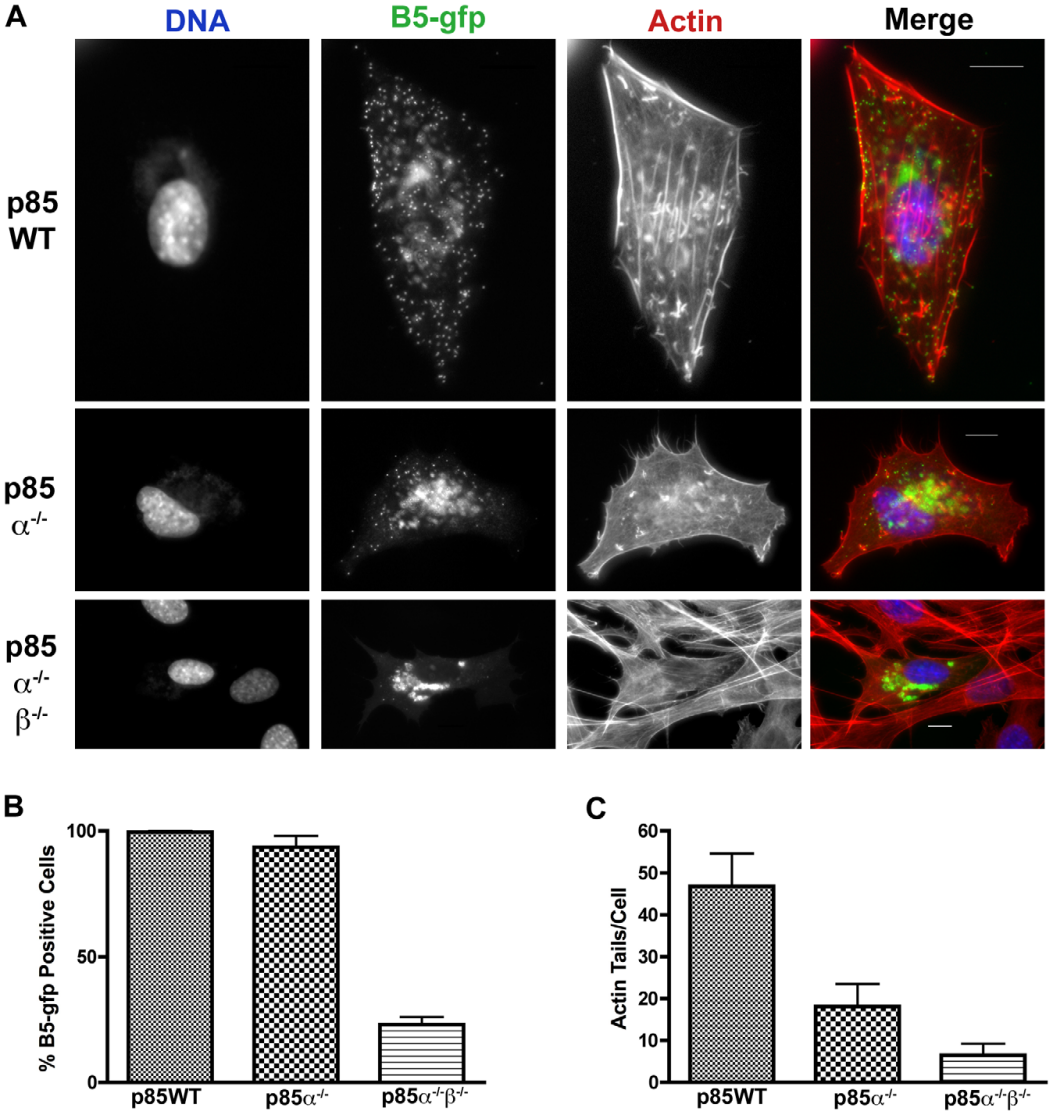
Localization of B5-GFP in p85-deficient cells. **A.** p85-deficient cells were infected with VV B5-GFP for 16 hours. Cells were fixed and stained with phalloidin to visualize actin (red) and DAPI to visualize DNA (blue). Note that in the p85α^−/−^ and p85α^−/−^β^−/−^ cells there is a reduction of punctate FITC fluorescence at the cell periphery, which represents virions, as well as a reduction in the number of actin tails. B5 remains in a peri-nuclear location in p85-deficient cells. **B.** p85-deficient cells were infected as in **A** and percent B5-GFP cells were quantified. Approximately 400 cells were counted per condition and cells were scored positive if a FITC (GFP) signal could be detected. p85α^−/−^β^−/−^ cells had significantly reduced rates of B5-GFP positive cells. Results are from three independent trials. **C.** p85-deficient cells form fewer actin tails than p85WT cells. Actin tails were counted for ∼10 cells per condition. Data is from one representative experiment.

Compared to wild type cells, p85α^−/−^ and p85α^−/−^β^−/−^ cells had fewer actin tails (Figure 6A, 6B, 7A, and 7C), and fewer B5-GFP- or F13-GFP fluorescent puncta in the cellular periphery (e.g. Figure 6A and 7A, lower panels). When evident, B5-GFP and F13-GFP fluorescence localized in a perinuclear region (Figures 6A and 7A, S3, Movies S4 and S5). However, the percentage of cells expressing detectable B5-GFP fluorescence was markedly reduced in p85α^−/−^β^−/−^ cells (Figure 7B). No significant difference in the amount or localization of the L1 protein was evident (Figure 6B) as measured by cellular fluorescence. Collectively, these observations indicate that PI3Ks mediate the localization of F13 and B5, and levels of L1, though some differences between the inhibitors and the p85-deficient cells were evident.

### PI3Ks regulate production of IMV and the amount of released virus

**Figure 8:**
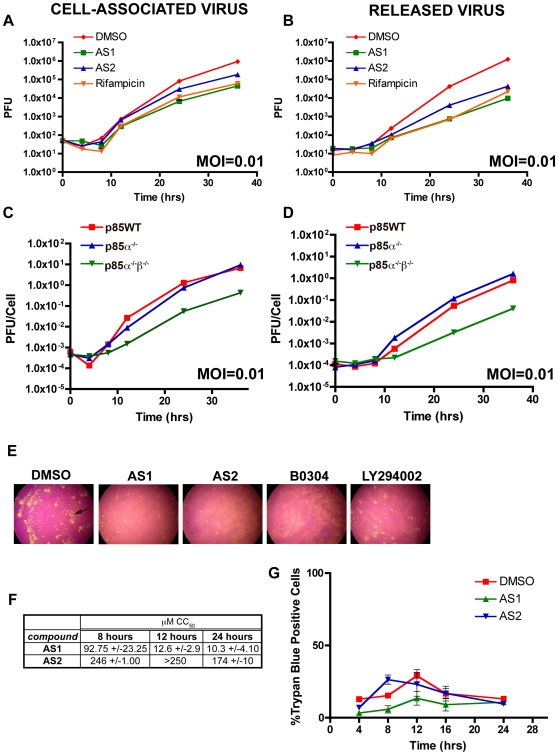
PI3K absence or inhibition reduces IHD-J virion production and release. **A-B.** Multistep growth curves of cell-associated (A) or released virus (B) conducted at an MOI of 0.01. AS1 and AS2 were used at 50 µM, Rifampicin 0.1 mg/mL, and the carrier DMSO at 0.5%. **A.** Multistep growth curves of cell-associated infectious virions, MOI of 0.01. AS1 and rifampicin reduce the amount of virus produced over time relative to DMSO control cells, (20.8-fold and 15.4-fold reduction at 36 hours). AS2 caused a modest reduction in the amount of virus produced over time relative to DMSO controls (5-fold reduction at 36 hours). **B.** Multistep growth curves of released infectious virions, MOI of 0.01. Note that AS1, AS2, and rifampicin reduce the amount of virus released into the supernatant (Fold reduction at 36 hours: AS1- 127.8-fold, AS2- 28.8-fold, Rifampicin- 55.6-fold). **C-D.** Multistep growth curves of cell-associated or released virus conducted at an MOI of 0.01 in p85-deficient cells. **C.** Multistep growth curves of cell-associated infectious virions, MOI of 0.01. From 8 to 36 hours, a reduction in the amount of virus produced was apparent in p85α^−/−^β^−/−^ cells compared to the p85WT and p85α^−/−^ cells (19.3-fold reduction at 36 hours). **D.** Multistep growth curves of released infectious virions, MOI of 0.01. A reduction in the amount of virus released from the p85α^−/−^β^−/−^ cells compared to the p85WT and p85α^−/−^ cells was apparent (20-fold reduction at 36 hours). Results are from one trial conducted in triplicate, and match growth curves conducted using WR. **E.** PI3K inhibitors reduce comet tails formed by VV, strain IHD-J. Monolayers were infected with 100PFU of virus, and plaques were visualized 48 hours later with crystal violet stain. Experiments were conducted in three independent trials. **F,G.** Toxicity assays of PI3K inhibitors on uninfected cells. **F.** MTS assay on BSC-1 cells treated with PI3K inhibitors for different periods of time. MTS production is a measure of mitochondrial metabolism. Values represent the concentration of drug (in µM) required to reduce formazan production by half. Experiments were conducted in two independent trials. **G.** Trypan blue exclusion assays of BHK cells treated with PI3K inhibitors (AS1 and AS2, 50 µM) for different time periods.

Based on the immunofluorescence microscopy, we hypothesized that PI3Ks may control formation of IMV and the wrapping of IEV/EEV. To assess viral production and released virus, we constructed growth curves at high and low MOI in the presence or absence of PI3K inhibitors. For all of these experiments, inhibitors were added one hour post adsorption to eliminate possible effects of PI3Ks on entry. At an MOI of 0.01 (Figure 8A), AS1 decreased the amount of cell-associated virus (CAV) relative to the DMSO controls by 20.8-fold. These effects appear similar to those observed with rifampicin (0.1 mg/mL), which blocks the morphogenesis of IMV and reduced viral loads by 15.4-fold (Figure 8A, see also [74]). In addition to the inhibitory effects on CAV production, AS1 also reduced the amount of virus released from cells by 127.8-fold (Figure 8B). AS2 treatment moderately reduced the production of CAV by 5-Fold (Figure 8A) and the amount of released virus by 28.8-fold (Figure 8B). No significant difference was evident in the amount of cell-associated and released virus produced in the p85WT and p85α^−/−^ cells at an MOI of 0.01 (Figure 8C and 8D). By contrast, p85α^−/−^β^−/−^ cells produced 19.3-fold less CAV, and released 20-fold less EEV (Figure 8C and 8D). For both the PI3K inhibitor-treated and p85-deficient cells, similar fold decreases were observed in virion production and release at MOIs ranging from 0.0001 to 5 (Figures S4A-D and S5A-D), suggesting that the observed effects reflected a role for PI3Ks in morphogenesis rather than cell-to-cell spread. Moreover, these data suggest that the block of virion morphogenesis by the PI3K inhibitors is incomplete, and it cannot be overcome simply by adding more virus to cells.

Another measure of released virus is the comet, an archipelago of small plaques that are evident adjacent to the main plaque (arrow in Figure 8E, DMSO) and have been attributed to the release of the EEV [69], [75]. Comets are particularly apparent with the poxvirus strain IHD-J [76]. Upon infection of cells with IHD-J and treatment with 50 µΜ AS2 or 20 µΜ B0304, plaques still form but comets are not evident (Figure 8E and Figure S6). By contrast, 50 µΜ AS1 and 20 µΜ LY294002 reduced the overall plaque size but comets are still visible, albeit smaller than those evident on control cells.

### PI3K inhibitors have limited toxicity

We considered the possibility that the drugs may have deleterious effects on cells. Therefore, we conducted toxicity assays during the same time periods that were used for the growth curves. Specifically, we measured the effect of the PI3K inhibitors on cellular respiration using an MTS assay (Figure 8F) and on cell viability using a trypan blue exclusion assay (Figure 8G). The MTS assay was conducted on uninfected BSC-1 cells at different drug concentrations for 8, 12, and 24 hours to calculate the amount of drug needed to reduce formazan production by half (CC_50_). Although the MTS assay indicated some cellular toxicity at high concentrations, we found that the cells remained impermeable to trypan blue dye (Figure 8G). Thus, although PI3K inhibitors may, at high concentrations disrupt mitochondrial enzymes, they do not appear to disrupt the cellular membrane. A TUNEL assay conducted at 16 hours post drug addition confirmed that the PI3K inhibitor treated cells and p85-deficient cells did not have significantly increased the rates of apoptosis (Figure S1A and S1B). Together, these data indicate that the compounds did not have significant toxic effects at the concentrations used to inhibit viral phenotypes and at exposure times relevant to this study. Moreover, the presence of plaques, albeit small ones, in the presence of drug suggests that inhibition of morphogenesis is not complete at the concentrations tested.

### PI3K inhibitors regulate virion morphogenesis at two distinct stages

**Figure 9:**
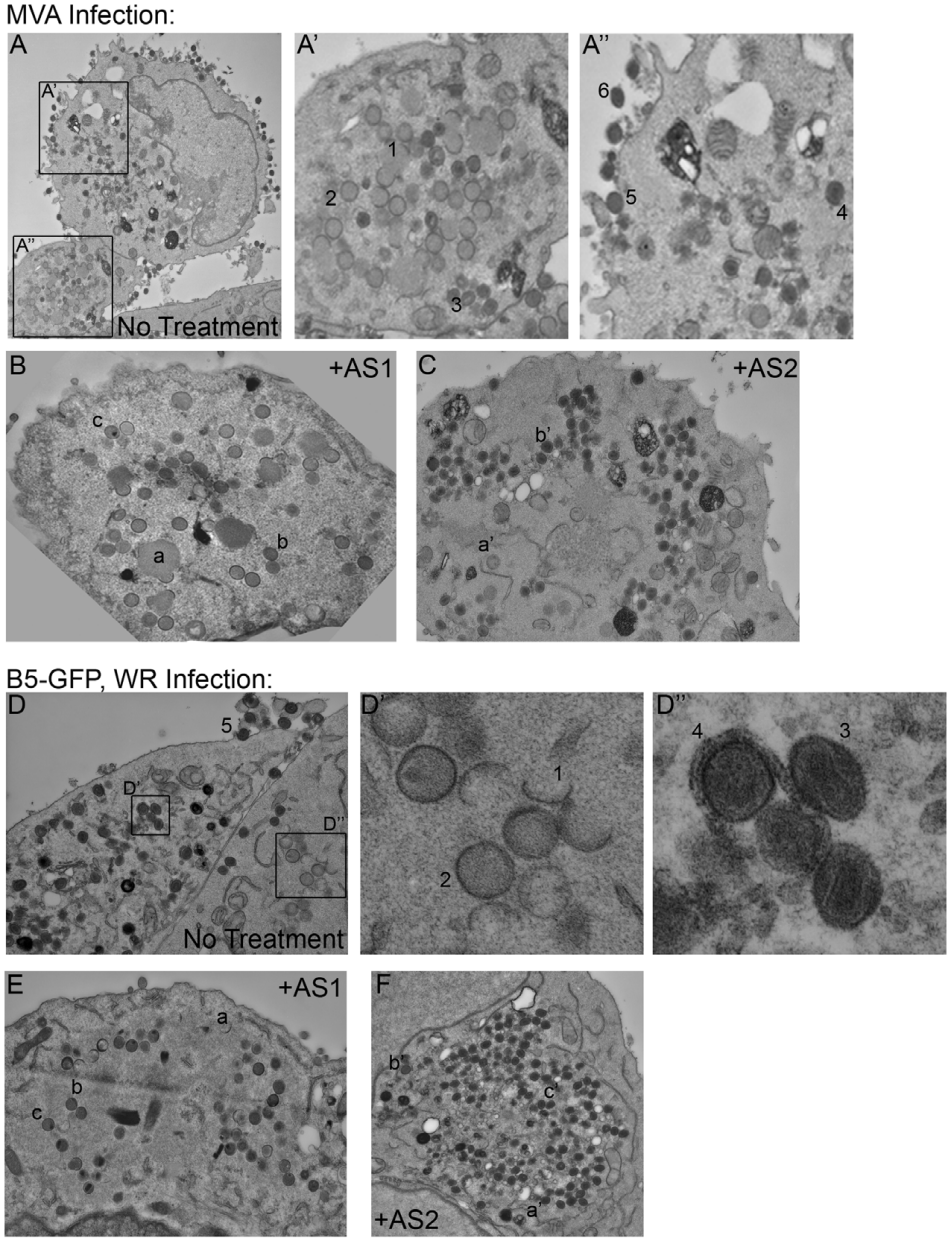
PI3K inhibitors disrupt vaccinia virus maturation. **A.** BHK cells were infected with MVA for 17 hours plus or minus PI3K inhibitors. Untreated samples exhibit characteristic virion morphogenesis (4100x, Glut/KMnO_4_ fixative). **A′:**1) Crescents; 2) Immature virions; 3) Mature virion, IMV; **A″:** 4) IEV; 5) CEV; 6) EEV. **B.** In cells treated with 50 µM AS1, virions at the immature to IMV transition, but not at later stages, are evident (10,000x, Glut/OsO_4_ fixative). a) Replication center and crescents; b) Immature virion; c) Immature virion with nucleoid. **C.** In cells treated with 50 µM AS2, virions at the IMV wrapping step but not at later stages are evident (6800x, Glut/KMnO_4_ Fix). a') Immature virion; b') IMV. Note the lack of virions associated with the plasma membrane, characteristic of IEV inhibition. **D.** HeLa cells were infected with B5-GFP WR for 17 hours plus or minus PI3K inhibitors. Untreated samples exhibit characteristic virion morphogenesis (10,000x, Glut/KMnO_4_ Fix). **D':** 1) Crescents; 2) Immature virions; **D″:** 3) Mature virion, IMV; 4) IEV. **E.** In cells treated with 50 µM AS1, virions at the immature to IMV transition, but not at later stages, are evident (10,000x, Glut/OsO_4_ Fix). a) Viral crescents; b) Immature virion; c) Immature virion with nucleoid. **F.** Samples treated with 50 µM AS2 are stuck at the IMV wrapping step (7000x, Glut/KMn_4_). a′) Crescents; b′) Immature virion; c′) IMV. Note the lack of virions associated with the plasma membrane, a characteristic of IEV inhibition.

To test the possibility that PI3K are directly involved in virion morphogenesis, we next visualized viral maturation by electron microscopy (EM) (Figure 9). Ultrastructural analysis of cells infected with the poxviruses MVA (Figure 9A-C) and B5-GFP, strain WR (Figure 9D-F) revealed virions at different stages of morphogenesis, as reported [31], [77]. These stages are annotated in Figure 9A and 9D and include 1) crescents; 2) immature virions (IV); 3) intracellular mature virions (IMV); 4) intracellular enveloped virions (IEV)); 5) cell-associated enveloped virions (CEV); and 6) extracellular enveloped virions (EEV). Treatment of cells with AS1 for 16 hours post-viral entry resulted in an apparent increase in the number of immature virions compared to untreated cells (Figure 9B and 9E). In addition, we could find few IMV. In contrast, AS2-treated cells had large numbers of IMV and EEV are not evident on the plasma membrane (Figure 9C and 9F). Additional images are presented in Figures S7, S8, S9, S10.

**Figure 10:**
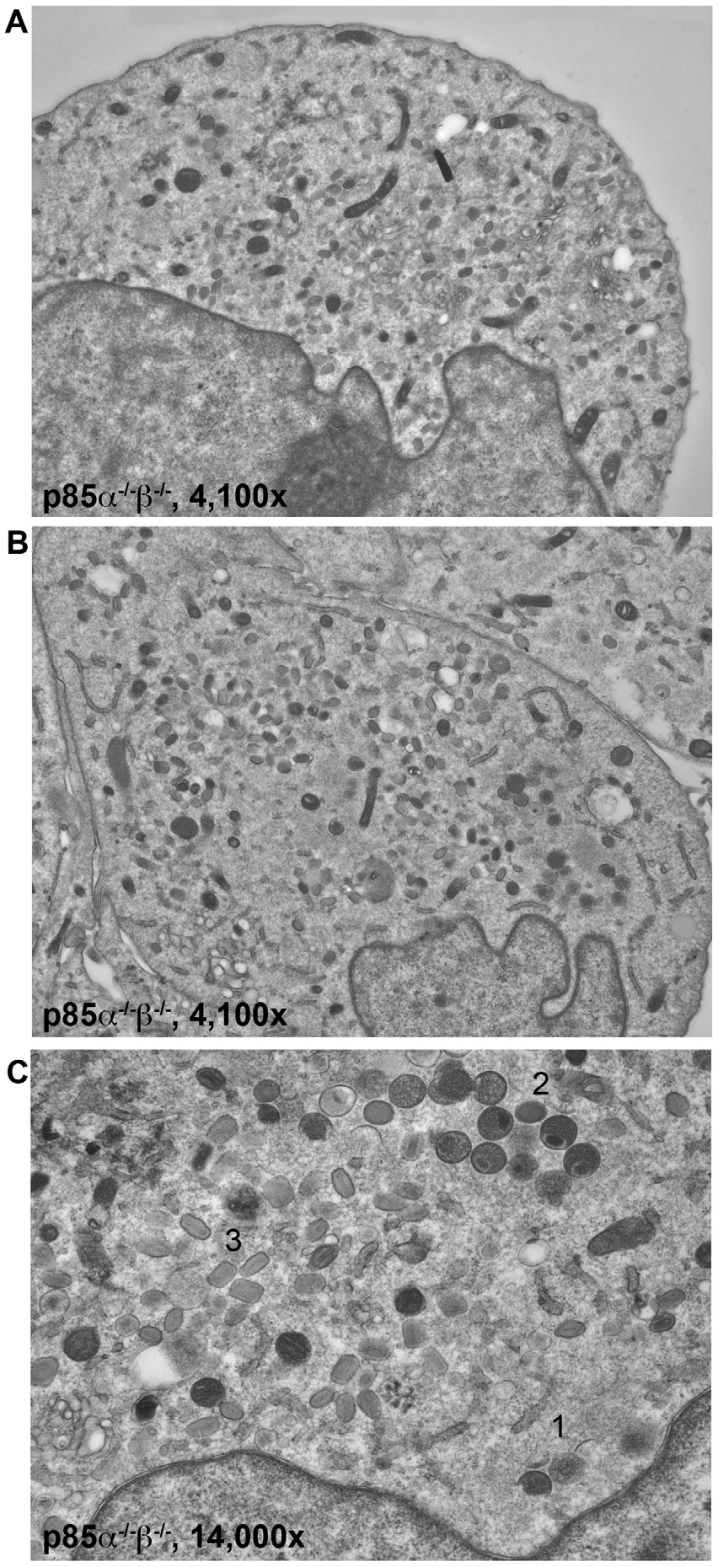
VV morphogenesis is disrupted in the p85-deficient cells. p85α^−/−^β^−/−^ cells were infected with B5-GFP WR for 17 hours. **A,B,C.** p85α^−/−^β^−/−^ cells exhibit characteristic virion morphogenesis (A,B. 4,100X, OsO_4_ Fix; C. 14,000X OsO_4_ fix), but are stuck at the IMV wrapping step. **1)** Crescents; **2)** Immature virions; **3)** Mature virion, IMV (4,100X, OsO_4_ Fix). Note the lack of virus associated with the plasma membrane.

Similar to the untreated BHK cells, the p85WT cells exhibited virions in multiple stages of maturation (not shown). p85α^−/−^β^−/−^ cells also had virions in multiple stages of maturation. These stages are annotated in Figure 10C and include 1) Crescents, 2) IV, and 3) IMV. However, in contrast to the p85WT cells, we were unable to find IEV in sections, and EEV are not evident on the plasma membrane of p85α^−/−^β^−/−^ cells (Figure 10A). Similar to the AS2-treated cells, we also observed large numbers of IMV (Figure 10B). Additional images are presented in Figures S11, S12, S13. Collectively these data suggest that PI3Ks regulate morphogenesis at two distinct transitions; IV to IMV transition (AS1-sensitive), and IMV envelopment to form IEV (AS2-sensitive, p85-dependent).

### PI3K usage by vaccinia virus is functionally redundant

**Figure 11:**
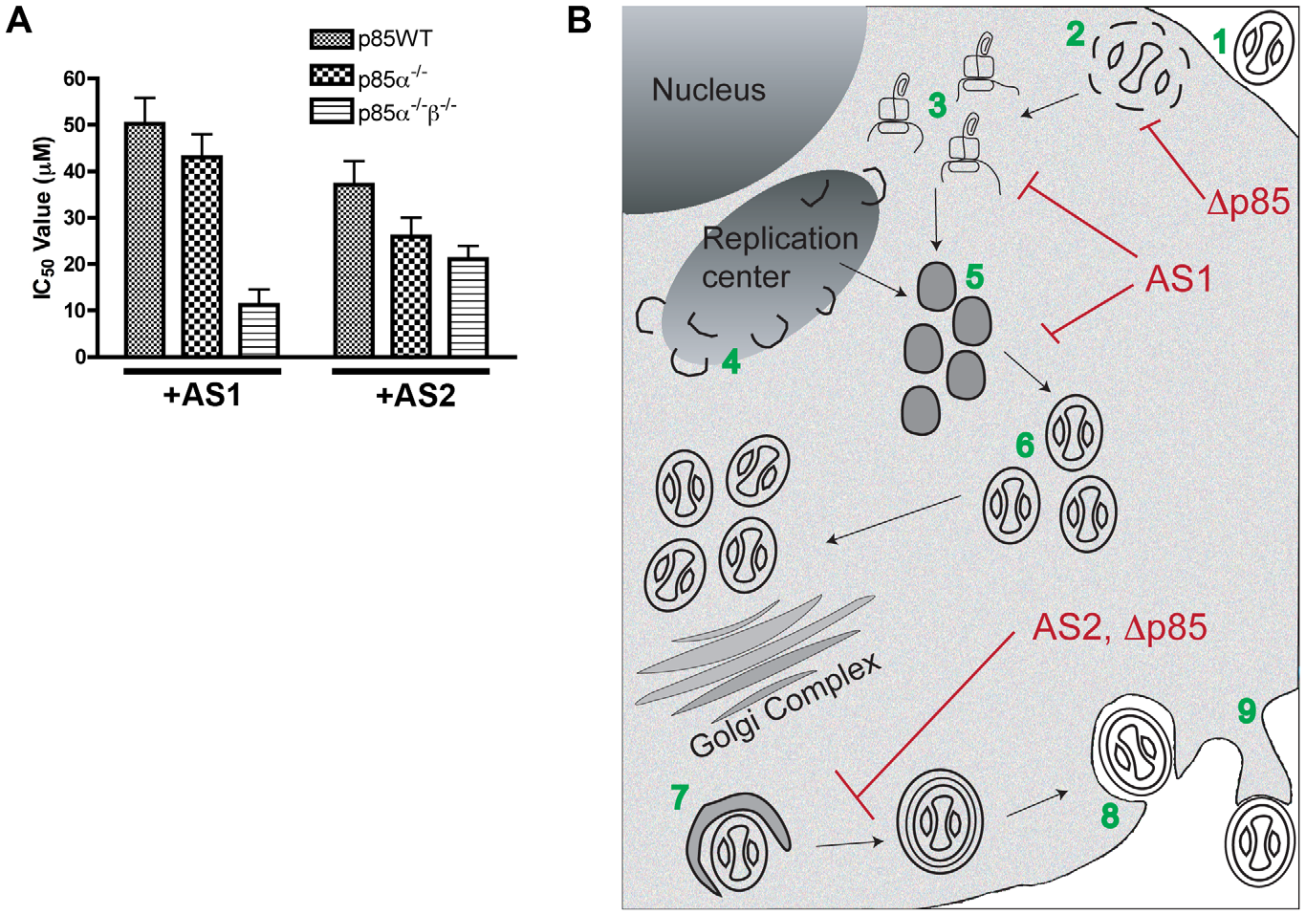
PI3K usage by VV is functionally redundant. **A.** PI3K inhibitors reduce plaque numbers in p85-deficient cells. Drugs were added to p85-deficient cells post viral adsorption at different concentrations. The concentration of drug needed to reduce plaque numbers by half (IC_50_) was calculated by fitting the data to a linear regression model using Prism software (GraphPad Prism Software, Inc., La Jolla, CA). IC_50_ values are lower for p85-deficient cells. Results are from three independent trials. **B.** Summary of vaccinia virus morphogenesis in p85-deficient cells or following PI3K inhibitor treatment. After VV entry (1) virion uncoating and early protein synthesis occurs (2). These steps are followed by late protein production (3), formation of viral replication centers and viral crescents (4). Crescents envelope viroplasm to form immature virions (IV, 5). The cores of IV condense to form mature virions (IMV, 6). A subset of the IMV traffic to the Golgi Complex where they are enveloped in a host cell derived membrane to form IEV (7). IEV fuse with the plasma membrane, releasing virus to the extracellular milieu, (CEV, 8). CEV form actin tails beneath the virus and ultimately, release to form extracellular enveloped virions (EEV, 9). Our results suggest that involvement of PI3Ks at steps 2,3,5, and 7. Steps 2 and 7 are disrupted in the p85-deficient cells, AS1 treatment disrupts steps 3 and 5, and AS2 disrupts steps 7. Thus, PI3K usage by VV is redundant; multiple PI3Ks acting at each of several steps regulate VV morphogenesis.

The observation that p85α^−/−^β^−/−^ cells exhibited only a slight reduction in virion production relative to wild-type cells (Figure 1B) suggests that VV may utilize multiple PI3K in a redundant fashion. To test this hypothesis, we assessed plaque formation in wild type and p85α^−/−^β^−/−^ cells in the presence or absence of PI3K inhibitors. In the p85α^−/−^ and p85α^−/−^β^−/−^ cells a significant fraction of type 1 PI3K activity is reduced [50], [66], however, the type 1b, type 2 and type 3 PI3Ks are still functional. We reasoned that a lower concentration of inhibitor would be required to block plaque formation in knockout cells compared to wild type cells. As shown in Figure 11A, the IC_50_ values, calculated on the basis of percent reduction in plaque number, were significantly higher for AS1 in p85WT cells (50.3 µM) compared to p85α^−/−^β^−/−^ cells (11.2 µM). A similar result was obtained for AS2 (37.1 µM) in p85WT cells compared to the p85α^−/−^β^−/−^ cells (21.1 µM). Together these data suggest that several PI-3 kinase isoforms, likely acting in a redundant fashion, regulate VV morphogenesis at multiple steps.

## Discussion

Herein we define the involvement of PI3Ks in orthopoxvirus morphogenesis using PI3K inhibitors and cell lines deficient in the regulatory subunit of Type I PI3Ks, p85. The inhibitors AS1 and AS2 were originally developed to inhibit the p110-gamma subunit of the Type Ib PI3K, but they also inhibit the other PI3K subtypes at higher concentrations [61], preventing assignation of a specific PI3K isoform to stages of orthopoxviral morphogenesis. Nevertheless, data with cell lines lacking p85 indicates that the PI3K inhibitors are likely targeting a host kinase and not a viral one. The inhibitors tested decrease plaque size for VV, ectromelia virus and monkeypox virus, implying that PI3K-dependent mechanisms are conserved among poxviruses. We hypothesize that differences in the IC_50_ and IC_20_ values for these viruses may be due to differential PI3K usage by the viruses, a topic that we are currently exploring. A summary of our results using the PI3K inhibitors and p85-deficient cells is presented in Figure 11B.

### PI3K contribution to early protein expression

We found that the PI3K inhibitors AS1 and AS2 when added post adsorption did not affect levels of early protein E3 (Figure 1C), whereas E3 levels were decreased in the p85 deficient cells independent of entry (Figure 1D and 1E, Figure 11B #2). Without EM, we cannot rule out the possibility that p85 deficient cells also have an entry defect. However, this seems unlikely because such an effect is not recapitulated in our growth curves (Fig. 8C and 8D, Figure S5). Thus our data suggest that PI3Ks that depend on p85 regulate levels of early proteins, but not virion entry. The reduction in early protein levels in the p85-deficient cells is in agreement with those obtained by Hu *et al*., demonstrating that early protein F4 levels are reduced with VV and Canarypox, following pretreatment with PI3K inhibitor LY294002 and in cells transiently transfected with a p85 dominant negative construct [78]. Work by Soares *et al*. is in partial agreement with this result; they observed a decrease in CrmA/SPI-2 levels following pretreatment with LY294002 and infection with Cowpox, but not VV [48]. Because both Hu *et al*. and Soares *et al*. pretreated cells with LY294002, it is possible that the observed defects in early protein synthesis do not result from effects on early protein synthesis, but rather result from defects in virion entry, a result reported by Mercer *et al*. [54]. By adding our compounds post viral adsorption we avoided such artifacts, and showed that early protein levels are reduced in a p85-dependent, but AS1/AS2-independent manner. However, we cannot rule out the possibility that adding of AS1 or AS2 at one hour following adsorption and entry was too late to affect a p85-dependent PI3K step that regulates early protein expression.

Although Assarsson *et al*. demonstrated that E3 is expressed early during an infection, no data exists describing the location of E3 transcription inside or outside the virion [79]. Because VV carries the entire complement of enzymes required for early gene transcription within the viral core, and early protein translation occurs after core uncoating, we hypothesize that p85-dependent PI3Ks may function in some aspect of core uncoating [80], [81], [82], [83]. Therefore, if viral uncoating is delayed, E3 transcription/translation may be delayed as well. Alternatively, Mallardo *et al.* have demonstrated that the sites of early protein synthesis are distinct from the sites of DNA replication [84]. Therefore, p85-dependent PI3Ks may also function to localize the early protein synthesis machinery.

### PI3K contribute to late protein expression

The observed defects in early protein levels in the p85-deficient cells have raised the possibility that delays in late protein synthesis can be ascribed to upstream effects on early proteins. Moreover, we cannot rule out the possibility that DNA replication may be delayed or diminished in these cells. However, P4b was still proteolytically processed, implying that the I7 protease was functional in these cells. We also found that AS1, but not AS2, appeared to reduce late proteins F13, B5, and P4b/4b to a similar extent as in the p85-deficient cells (Figure 11B, #3). Notably, AS1 and p85-deficient cells did not appear to exert a complete block of morphogenesis as P4b was still proteolytically processed and infectious virus was still produced and released in these cells. Nevertheless, inhibitors AS1 and AS2 both reduce plaque size to a similar degree, and our results suggest that they may be doing so by different mechanisms.

Both inhibitors were developed to inhibit the p110-gamma isoform of the PI3Ks and structurally, they have the same chemical backbone, but differ in side group substitutions. Interestingly, Camps *et al.* demonstrate that AS1 and AS2 inhibit the similar classes of PI3K isoforms, albeit with different efficacy [61]. We hypothesize that the differences in drug structure and kinase specificity lead to the observed differences in late protein synthesis. We cannot rule out the possibility that differences between the p85-deficient cells and PI3K-inhibitor treated cells may be attributed to p85-dependent, catalytic subunit–independent signaling, a mechanism that has been described previously [50], [85].

Zaborowska and Soares *et al.* also found that pretreatment of VV infected cells with LY294002 reduces late IMV protein production, a result consistent with our AS1 data [48], [67]. Zaborowska *et al.* demonstrate that this decrease is due to disrupted eIF4F complex following PI3K inhibition [67]. Furthermore, Hu *et al.* identified as similar reduction in late protein F4 mRNA levels following LY294002 pretreatment or in cells expressing a p85 dominant negative construct [78]. Our results with PI3K inhibitors and in p85 deficient cells are consistent with, but also expand and qualify these results. We demonstrate that AS1 not only reduces late IMV proteins, but also reduces late IEV/CEV/EEV-specific proteins as well (Figure 2). Furthermore, our data provides a context for the involvement of host PI3K in VV morphogenesis by tying these results to specific p85 isoforms.

### PI3K regulate B5 and F13 trafficking

Husain *et al.* demonstrated that F13 regulates B5 movement from the Golgi to post-Golgi vesicles [35]. Specifically, upon expression of F13 with mutations in the palmitylation site or with mutations in the phospholipase active site motif, both B5 and F13 remain in the cytoplasm or cis-Golgi (p115-positive) structures [35] and fail to localize to peripheral punctate structures. Roper and Sung further demonstrate that these same mutations in the phospholipase C motif reduce both plaque and IEV/CEV/EEV formation [72], [73]. Furthermore, butanol-1 treatment, which inhibits phospholipase D activity, also decreases EEV production and redistributes F13 to the juxtanuclear Golgi region [86]. Moreover, Ward *et al.* also showed that mutations in a dileucine motif of a chimeric B5 protein could prevent plasma membrane retrieval, as measured by antibody uptake and staining, implying a mechanism for B5 retrograde trafficking [87]. Altogether, these results demonstrate that F13 and B5 localization depends on endosomal trafficking, and given the potential phospholipase activity of F13, may also depend on phospholipid turnover.

Consistent with this idea, we found that localization of F13 and B5 was dependent on PI3K activity. AS1 treatment caused F13 to localize to a cytoplasmic compartment, in a manner reminiscent of F13(C186S) palmitylation and F13(D319E) phospholipase D (PLD) active site mutants, which exhibited similar localization [35]. In contrast, AS2 treatment caused B5 and F13 to remain in a perinuclear compartment, although “breakthrough” wrapped virus and actin tails were observed. The phenotype seen in the p85-deficient cells appears to be closer to that seen with AS2 treatment and resembles the F13(C185S) and F13(K314R) mutants, which remain in a cis-Golgi (p115-positive) compartment [35]. The phenotypic similarities of PI3K absence/inhibition with the palmitylation and PLD active site mutants demonstrates that F13 and B5 trafficking is dependent on membrane association and lipid signaling.

### PI3K mediate VV morphogenesis

Our data with PI3K inhibitors and p85-deficient cells suggest that morphogenesis is differentially regulated by multiple PI3Ks at different stages. Specifically, differences in virion production for both the PI3K inhibitors and the p85-deficient cells were evident in growth curves, by microscopy, and by electron microscopy, and indicate that PI3Ks regulate production of both IMVs (Figure 11B, #5) and IEV/EEVs (Figure 11B, #7). The AS1 inhibitor morphological block appeared reminiscent of morphological defects seen with the vaccinia redox proteins, A2.5, E10, G4, phosphoprotein A13 and LY294002 pretreatment [20], [22], [26], [28], [48]. In contrast, AS2-treated cells had large numbers of IMV (Figure 9C and 9F), reminiscent of morphological defects seen following IMCBH treatment [88], [89]. Similar to AS2-treated cells, p85 deficient cells did not wrap IMV to form IEV and viral morphogenesis in these cells appears similar to the AS2-treated cells. Furthermore, we observed similar morphological defects following PI3K inhibition/absence in both MVA and WR infected cells, suggesting that the involvement of host PI3K is conserved across multiple poxviral species. Overall, the similarity of viral morphological blocks seen in inhibitor treated cells and the p85-deficient cells demonstrates that PI3Ks function to correctly localize envelope-specific viral proteins to nascent envelopes, leading to the formation of the envelope membranes.

### VV Uses PI3K in a redundant manner

Several observations suggest that VV uses PI3K redundantly. We define redundancy as the capacity to use PI3K at multiple steps of virion morphogenesis and the ability to use multiple PI3K at each morphological step. In contrast to previous reports with particular PI3K inhibitors (e.g. LY294002 and Wortmannin), our use of several PI3K inhibitors has allowed us to resolve several stages at which PI3K act, including the IV to IMV transition and, the IMV to IEV transition. Furthermore, we observed that the p85α^−/−^β^−/−^ cells had delayed early protein synthesis and were deficient in IMV wrapping. These data suggest that p85-dependent PI3K and PI3K-inhibitor sensitive PI3K are used for multiple steps of virion morphogenesis.

Furthermore, we find that VV can use multiple PI3K at each morphological step, because plaque formation was only reduced by half in the p85α^−/−^β^−/−^ cells compared to the p85α^−/−^ and p85WT cells (Figure 1B). This incomplete phenotype lead us to hypothesize that PI3K activity persisted in the p85-deficient cells because the p85 regulatory subunit is only used by the type 1a PI3K kinases, and the type 1b, type 2 and type 3 phosphatidylinositol 3-kinases are still functional in the p85α^−/−^β^−/−^ cells. We found that addition of the PI3K inhibitors had an additive effect in reducing the number of plaques on the p85 deficient cells and could completely eliminate plaques at higher concentrations. Together, these observations suggest that VV uses PI3K in a functionally redundant manner; that is, VV uses multiple PI3Ks at each of several steps in morphogenesis. Our use of PI3K inhibitors in conjunction with p85-deficient cells provides a significant advance in our understanding of how PI3Ks are utilized by poxviruses.

PI3K redundancy may help to extend both the host range and cell type specificity for the virus, as PI3K isoforms are differentially expressed through out the body [37]. Such redundancy is not without precedent. Viable mice can be generated from mice missing one splice variant of p85α, but not from mice missing all p85α splice variants, implying that the p85α isoforms are functionally redundant [90]. Moreover, redundancy may also be a common feature of host-pathogen signaling. Swimm *et al.* and Reeves *et al.* demonstrate that EPEC and VV can use host tyrosine kinases redundantly to make actin protrusions [52], [91], [92]. Redundancy in PI3K signaling has also been reported for Ebola entry into p85-deficient cells [93], and insulin signaling in HepG2 cells [94].

### Why would VV use PI3Ks for virion morphogenesis?

Other viruses, including VV and cowpox, activate PI3K to inhibit cellular apoptosis [47], [48], [49]. However, other pathogens, like *Salmonella* and *M. tuberculosis*, disrupt PI3K signaling to inhibit endosome to lysosome fusion, thereby creating a novel pathogen-specific endosome [95], [96], [97]. PI3K signaling has been shown to act as a signaling molecule, creating phosphatidylinositol 3-phosphate (PI3P) microdomains on membranes [98]. The local PI3P concentration on endosomes can act as a “zipcode” within the host cells; for instance, PI3P is usually localized to multi-vesicular bodies, autophagosomes and early endosomes [99], [100]. We demonstrate that VV uses PI3Ks to correctly target and localize components required for morphogenesis. This may consist of both the protein components (e.g. B5 and F13) as well as lipid components, as the IMV form has been shown to be enriched in phosphoinositides [7], [101], [102]. PI3K interact with many of the rab proteins [103], [104], and rab1 and rab2 are markers for the intermediate compartment [105] and have already been shown to co-localize with VV replication centers [7], [106], perhaps suggesting a mechanism for PI3K-dependent acquisition of the IMV envelope acquisition.

PI3K may also regulate IMV wrapping to form IEV. Chen *et al.* provide evidence that F13 can interact with host proteins TIP47 and rab9 to nucleate the IMV wrapping complex [107]. Membranes derived from early endosomes can also envelope IMV particles, as Tooze *et al.* demonstrate that cells treated with Brefeldin A still envelope IMV, and that these IEV are positive for exogenously added HRP [30]. Mechanistically, PI3K inhibitors or deficient cells could disrupt IMV wrapping by interfering with Golgi and/or early endosome trafficking. Since other enveloped viruses need to acquire a lipid membrane, it is possible that PI3Ks and lipid dynamics may be utilized by other wrapped viruses to highjack host cell membranes. Overall, the small molecules that target PI3Ks or other host enzymes that regulate morphogenesis yield new insight into the mechanism of viral envelopment and may represent a new class of antiviral therapeutic drugs, a prospect that we are currently testing.

## Supporting Information

Table S1 20% inhibitory concentration (IC_20_) of PI3K inhibitors added post-adsorption to poxvirus-infected monolayers. 1[63] 2[113] 3[67]
(0.03 MB DOC)

Figure S1 S1A. TUNEL assay for PI3K inhibitor treated BHK cells. PI3K inhibitors were added to uninfected BHK cells on glass coverslips for 16 hours. Media was removed and the cells were fixed and processed for the TUNEL assay. AS1 and AS2 were added at 50 µM and were in 0.5% DMSO. B0304 and LY294002 were at 20 µM and were in 0.2% DMSO. DNase1 was used as a positive control. Approximately 700 cells were counted per condition. PI3K inhibitors did not increase the levels of apoptosis in the BHK cells following 16 hour treatment. S1B. TUNEL assay for p85-deficient cells. Uninfected p85WT or -deficient cells were added to glass coverslips for 16 hours, and then fixed and processed for the TUNEL assay. DNase1 was used as a positive control. Approximately 700 cells were counted per condition. The rates of apoptosis are not increased in the p85-deficient cells.
(0.23 MB TIF)

Figure S2 PI3K inhibitors reduce the number of actin tails/cell. BSC40 cells were infected with WR and treated with 100 µM AS1 and AS2 post-adsorption, and the cells were fixed and stained with FITC-phalloidin 16 hours later. The number of actin tails was counted on ten cells per condition.
(0.75 MB TIF)

Figure S3 Screen shots from z-stack of spinning disk microscopy of p85WT or p85α^−/−^β^−/−^ cells infected with F13-GFP. Note the punctate virions in the p85WT cells, and the lack of these structures in the p85α^−/−^β^−/−^ cells. Instead, the F13 protein localizes to a peri-nuclear structure.
(1.09 MB TIF)

Figure S4 A-B. Single step growth curves conducted at MOI = 5 in PI3K inhibitor-treated cells. VV, strain IHD-J, was allowed to bind and enter BHK cells for 1 hour, monolayers were washed with PBS twice, and media was added containing PI3K inhibitors (50 µM), DMSO (0.5%) or Rifampicin (0.1 mg/mL). C-D. Multistep growth curves conducted in PI3K inhibitor-treated cells. Cells were infected at different MOIs with Vaccinia virus, strain IHD-J. Virus was allowed to bind and enter BHK cells for 1 hour, monolayers were washed with PBS twice, and media was added containing PI3K inhibitors (50 µM), DMSO (0.5%) or Rifampicin (0.1 mg/mL). Supernatants and monolayers were collected at 24 hours post infection.
(0.68 MB TIF)

Figure S5 A-B. Single step growth curves conducted at MOI = 5 in p85-deficient cells. VV strain IHD-J was allowed to bind and enter cells for 1 hour, monolayers were washed with PBS twice, and fresh media was added. C-D. Multistep growth curves conducted in p85-deficient cells. Cells were infected at different MOIs with VV strain IHD-J as in A and B.
(0.63 MB TIF)

Figure S6 PI3K inhibitors reduce comet tails formed by VV, strain IHD-J. Insets show enlarged image of plaques boxed in top panels. Insets are at the same scale.
(2.20 MB TIF)

Figure S7 PI3K inhibitors disrupt vaccinia virus maturation. BHK cells were infected with MVA for 17 hours and treated with 50 µM AS1 (8,200X, KMnO_4_ fix).
(9.91 MB PSD)

Figure S8 PI3K inhibitors disrupt vaccinia virus maturation. BHK cells were infected with MVA for 17 hours and treated with 50 µM AS1 (8,200X OsO_4_ Fix).
(9.59 MB PSD)

Figure S9 PI3K inhibitors disrupt vaccinia virus maturation. HeLa cells were infected with B5-GFP for 17 hours and treated with 50 µM AS2 (8,200X, KMnO_4_ fix).
(5.22 MB TIF)

Figure S10 PI3K inhibitors disrupt vaccinia virus maturation. HeLa cells were infected with B5-GFP for 17 hours and treated with 50 µM AS2 (8,200X, KMnO_4_ fix).
(5.30 MB TIF)

Figure S11 VV morphogenesis is disrupted in the p85-deficient cells. p85α^−/−^β^−/−^ cells were infected with B5-GFP WR for 17 hours (2,700X, OsO_4_ Fix).
(3.45 MB TIF)

Figure S12 VV morphogenesis is disrupted in the p85-deficient cells. p85αα^−/−^ β^−/−^ cells were infected with B5-GFP WR for 17 hours (4,100X, OsO_4_ fix).
(5.47 MB TIF)

Figure S13 VV morphogenesis is disrupted in the p85-deficient cells. p85α^−/−^β^−/−^ cells were infected with B5-GFP WR for 17 hours. (8,200X OsO_4_ fix).
(5.07 MB TIF)

Movie S1 Spinning disk microscopy of untreated HeLa cells infected with VV B5-GFP strain WR for 17 hours.
(2.55 MB MOV)

Movie S2 Spinning disk microscopy of HeLa cells infected with VV B5-GFP strain WR and treated with AS1 50 µM for 17 hours.
(1.68 MB MOV)

Movie S3 Spinning disk microscopy of HeLa cells infected with VV B5-GFP strain WR and treated with AS2 50 µM for 17 hours.
(2.26 MB MOV)

Movie S4 Spinning disk microscopy of p85WT cells infected with VV F13-GFP for 17 hours.
(7.92 MB MOV)

Movie S5 Spinning disk microscopy of p85α^−/−^β^−/−^ cells infected with VV F13-GFP for 17 hours.
(13.68 MB MOV)
