# Supplementary material for: Multiple Phosphatidylinositol 3-Kinases Regulate Vaccinia Virus Morphogenesis
Source: PLoS One. 2010 May 28;5(5):e10884. doi: 10.1371/journal.pone.0010884 (PMC2878334; doi:10.1371/journal.pone.0010884)
Supplement: Table S1 — 20% inhibitory concentration (IC20) of PI3K inhibitors added post-adsorption to poxvirus-infected monolayers. 1[63] 2[113] 3[67] (0.03 MB DOC) [file pone.0010884.s001.doc]

Supplementary Table 1:

|  | **Inhibits:** | **Vaccinia IC_20_** | **Ectromelia IC_20_** | **Monkeypox IC_20_** | **Other Targets:** |
| --- | --- | --- | --- | --- | --- |
| **AS1** | p110-gamma | 16.32 ±1.9μM | 6.87 ±1.5μM | 4.56 ±4.7μM | p110-alpha, p110-beta, p110-delta, PKCbII^1^ |
| **AS2** | p110-gamma | 21.79 ±3.1μM | 27.00 ±3.4μM | 3.58 ±1.4μM | p110-alpha, p110-beta, p110-delta^1^ |
|  |  |  |  |  |  |
| **B0304** | p110-alpha | 4.00 ±0.3μM | 3.25 ±0.2μM | 0.8 ±0.6μM | p110-beta, p110-delta, p110gamma^2^ |
| **LY294002** | Broad-Spectrum PI3K Inhibitor^3^ | 19.76 ±6.4μM | 1.05 ±0.0μM | 2.83 ±3.0μM |  |
