# Supplementary figures and images for: Multiple Phosphatidylinositol 3-Kinases Regulate Vaccinia Virus Morphogenesis
Source: PLoS One. 2010 May 28;5(5):e10884. doi: 10.1371/journal.pone.0010884 (PMC2878334; doi:10.1371/journal.pone.0010884)

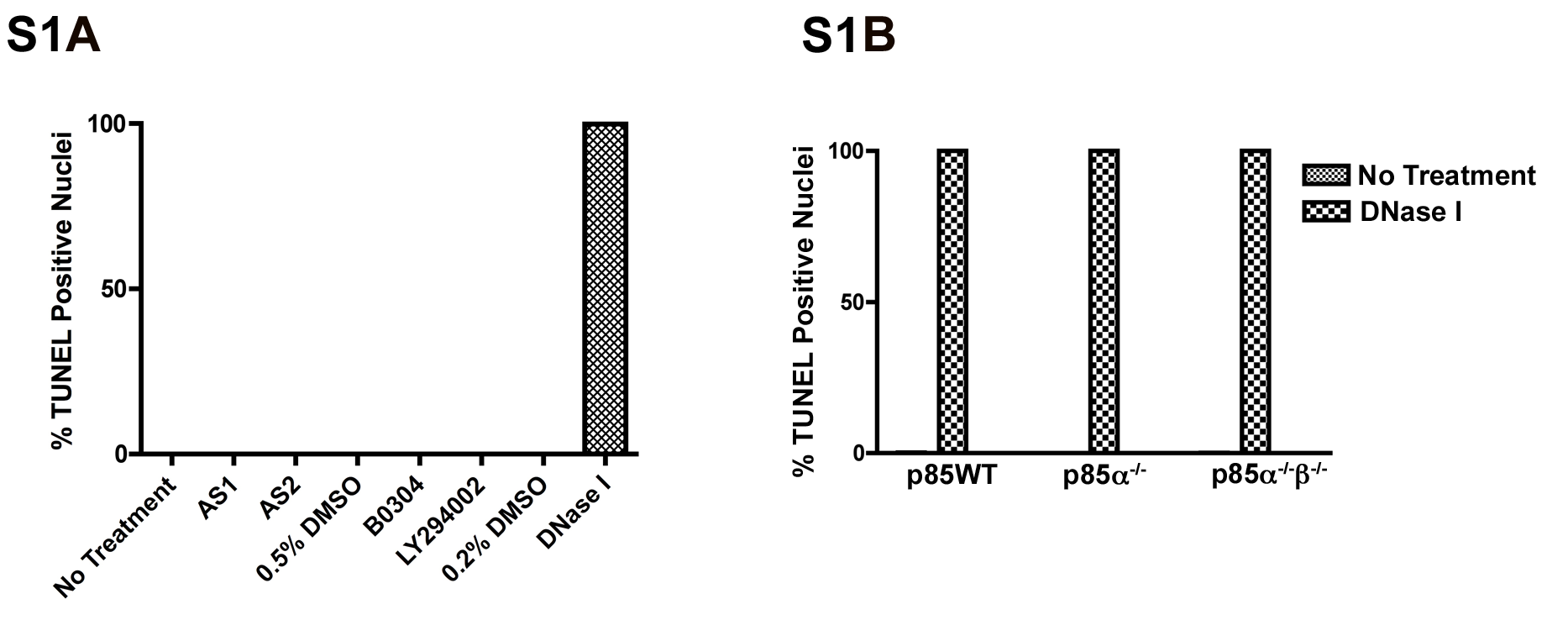

Supplement: Figure S1 — S1A. TUNEL assay for PI3K inhibitor treated BHK cells. PI3K inhibitors were added to uninfected BHK cells on glass coverslips for 16 hours. Media was removed and the cells were fixed and processed for the TUNEL assay. AS1 and AS2 were added at 50 µM and were in 0.5% DMSO. B0304 and LY294002 were at 20 µM and were in 0.2% DMSO. DNase1 was used as a positive control. Approximately 700 cells were counted per condition. PI3K inhibitors did not increase the levels of apoptosis in the BHK cells following 16 hour treatment. S1B. TUNEL assay for p85-deficient cells. Uninfected p85WT or -deficient cells were added to glass coverslips for 16 hours, and then fixed and processed for the TUNEL assay. DNase1 was used as a positive control. Approximately 700 cells were counted per condition. The rates of apoptosis are not increased in the p85-deficient cells. (0.23 MB TIF) [file pone.0010884.s002.tif]

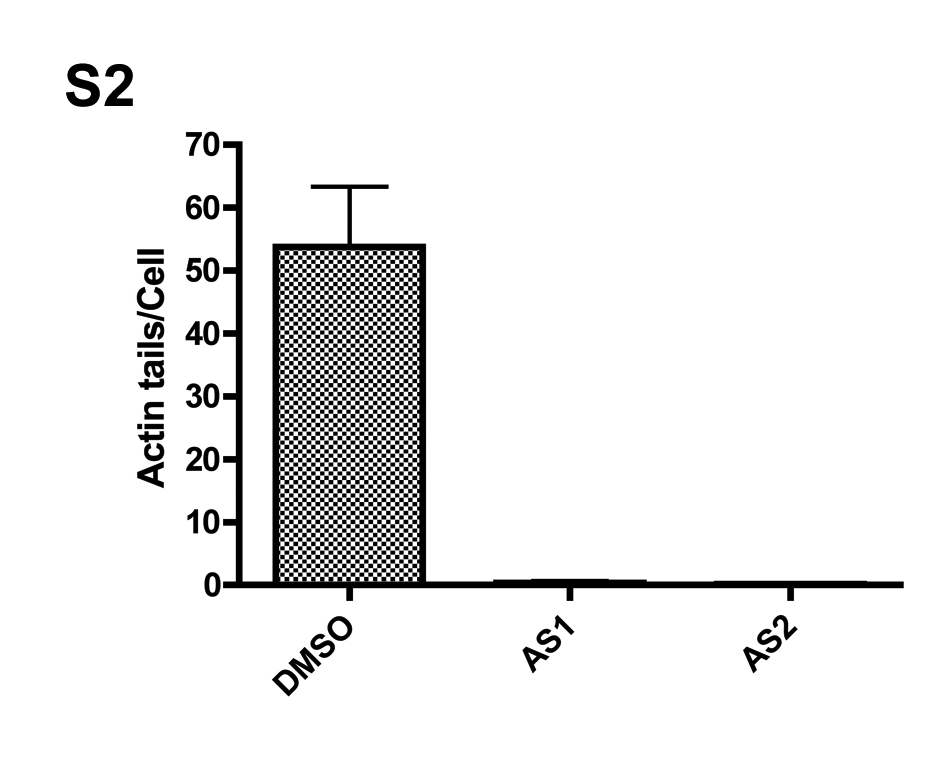

Supplement: Figure S2 — PI3K inhibitors reduce the number of actin tails/cell. BSC40 cells were infected with WR and treated with 100 µM AS1 and AS2 post-adsorption, and the cells were fixed and stained with FITC-phalloidin 16 hours later. The number of actin tails was counted on ten cells per condition. (0.75 MB TIF) [file pone.0010884.s003.tif]

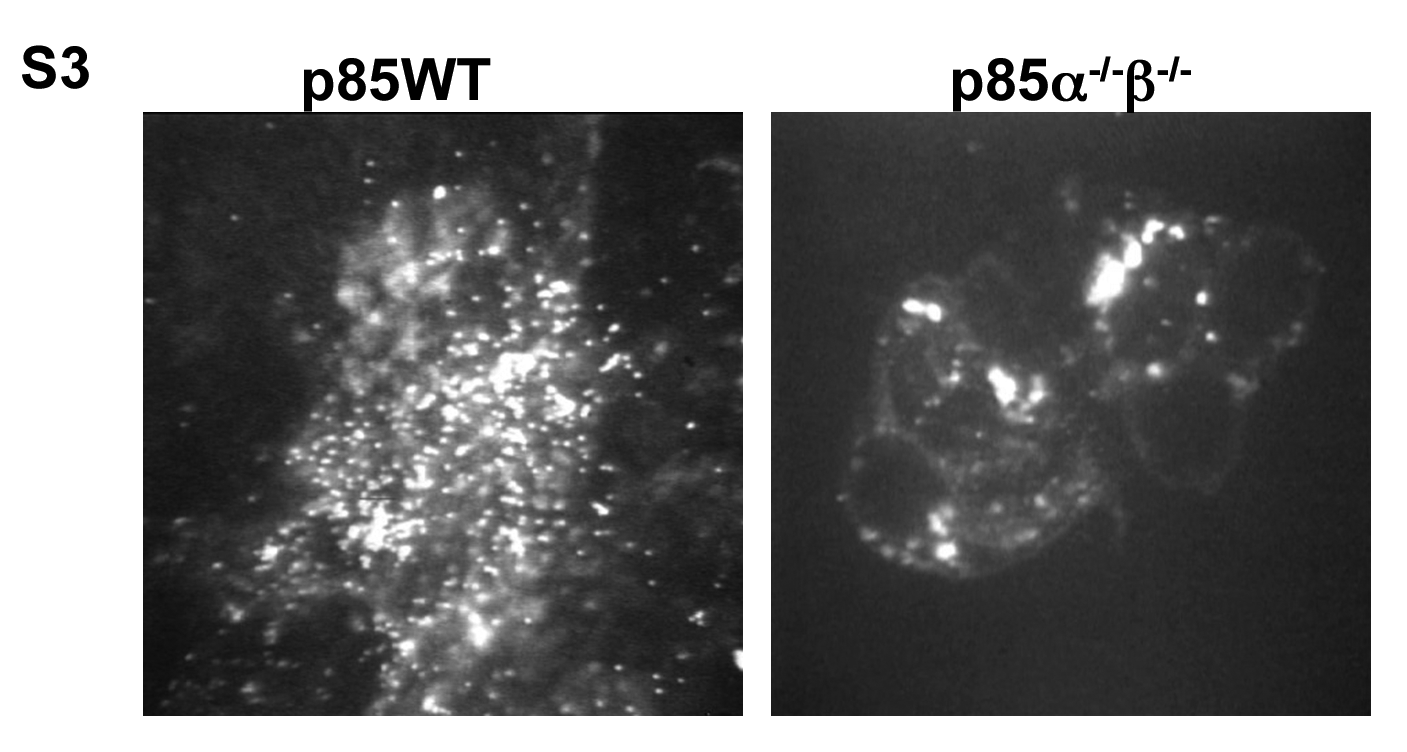

Supplement: Figure S3 — Screen shots from z-stack of spinning disk microscopy of p85WT or p85α−/−β−/− cells infected with F13-GFP. Note the punctate virions in the p85WT cells, and the lack of these structures in the p85α−/−β−/− cells. Instead, the F13 protein localizes to a peri-nuclear structure. (1.09 MB TIF) [file pone.0010884.s004.tif]

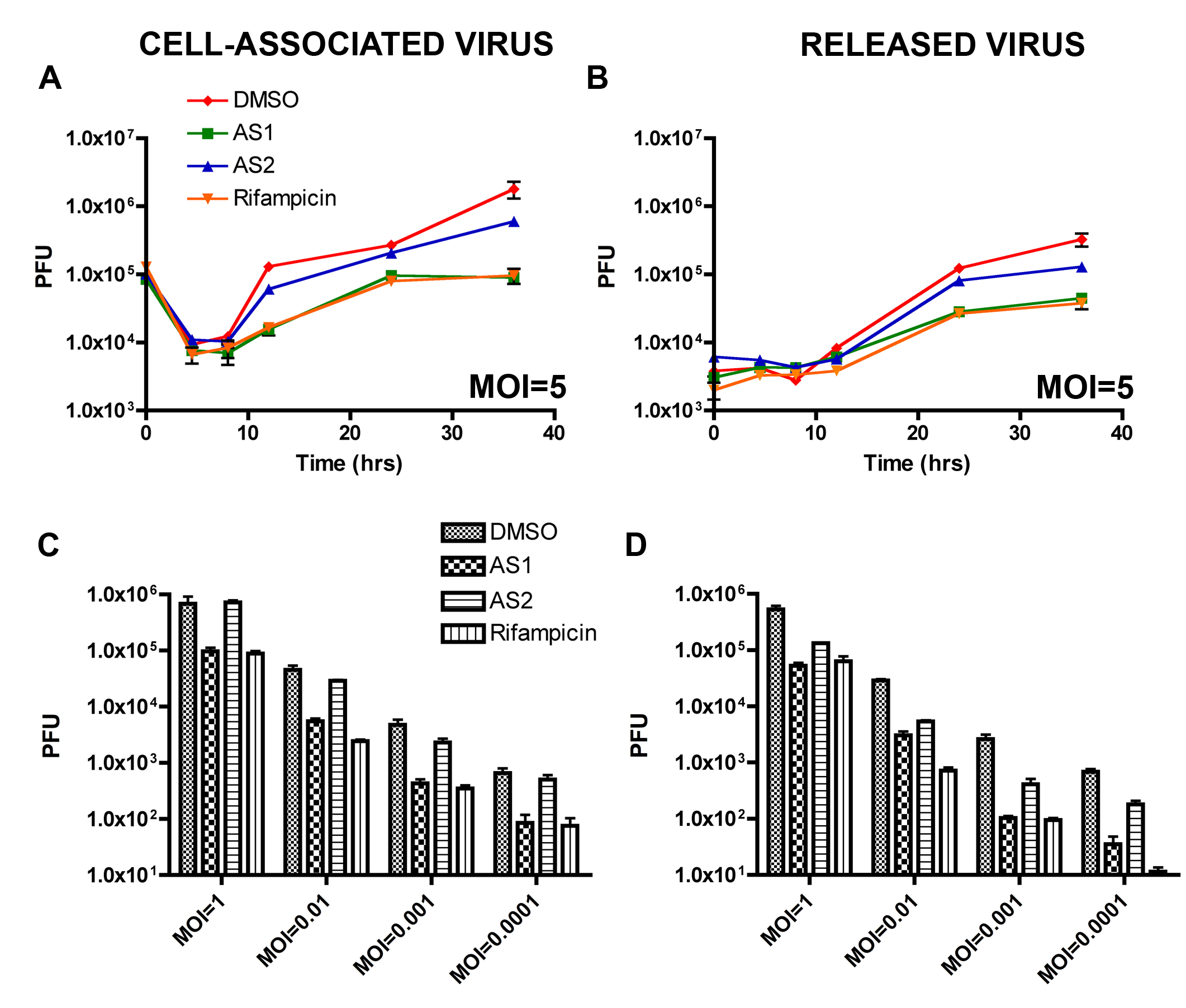

Supplement: Figure S4 — A-B. Single step growth curves conducted at MOI = 5 in PI3K inhibitor-treated cells. VV, strain IHD-J, was allowed to bind and enter BHK cells for 1 hour, monolayers were washed with PBS twice, and media was added containing PI3K inhibitors (50 µM), DMSO (0.5%) or Rifampicin (0.1 mg/mL). C-D. Multistep growth curves conducted in PI3K inhibitor-treated cells. Cells were infected at different MOIs with Vaccinia virus, strain IHD-J. Virus was allowed to bind and enter BHK cells for 1 hour, monolayers were washed with PBS twice, and media was added containing PI3K inhibitors (50 µM), DMSO (0.5%) or Rifampicin (0.1 mg/mL). Supernatants and monolayers were collected at 24 hours post infection. (0.68 MB TIF) [file pone.0010884.s005.tif]

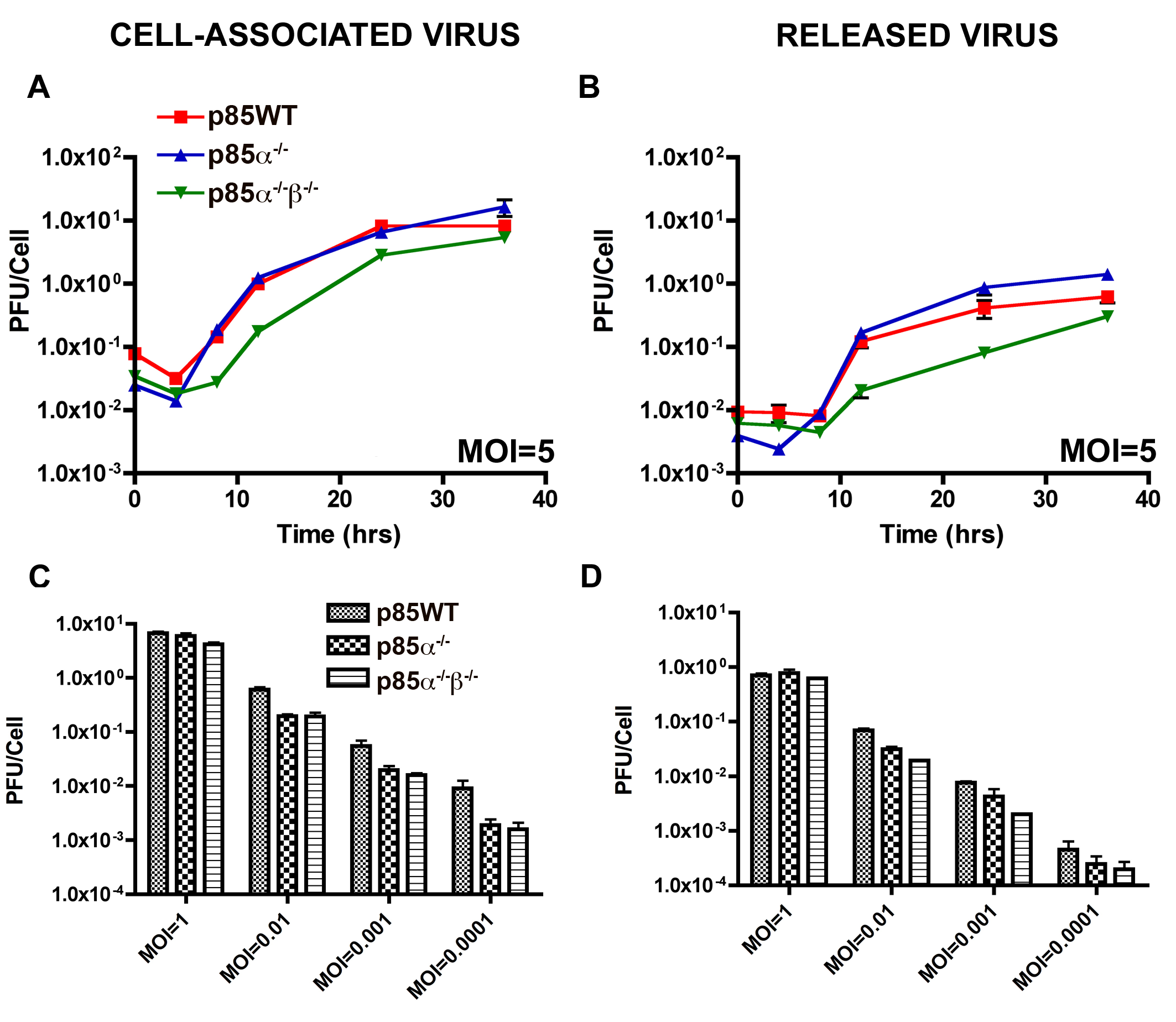

Supplement: Figure S5 — A-B. Single step growth curves conducted at MOI = 5 in p85-deficient cells. VV strain IHD-J was allowed to bind and enter cells for 1 hour, monolayers were washed with PBS twice, and fresh media was added. C-D. Multistep growth curves conducted in p85-deficient cells. Cells were infected at different MOIs with VV strain IHD-J as in A and B. (0.63 MB TIF) [file pone.0010884.s006.tif]

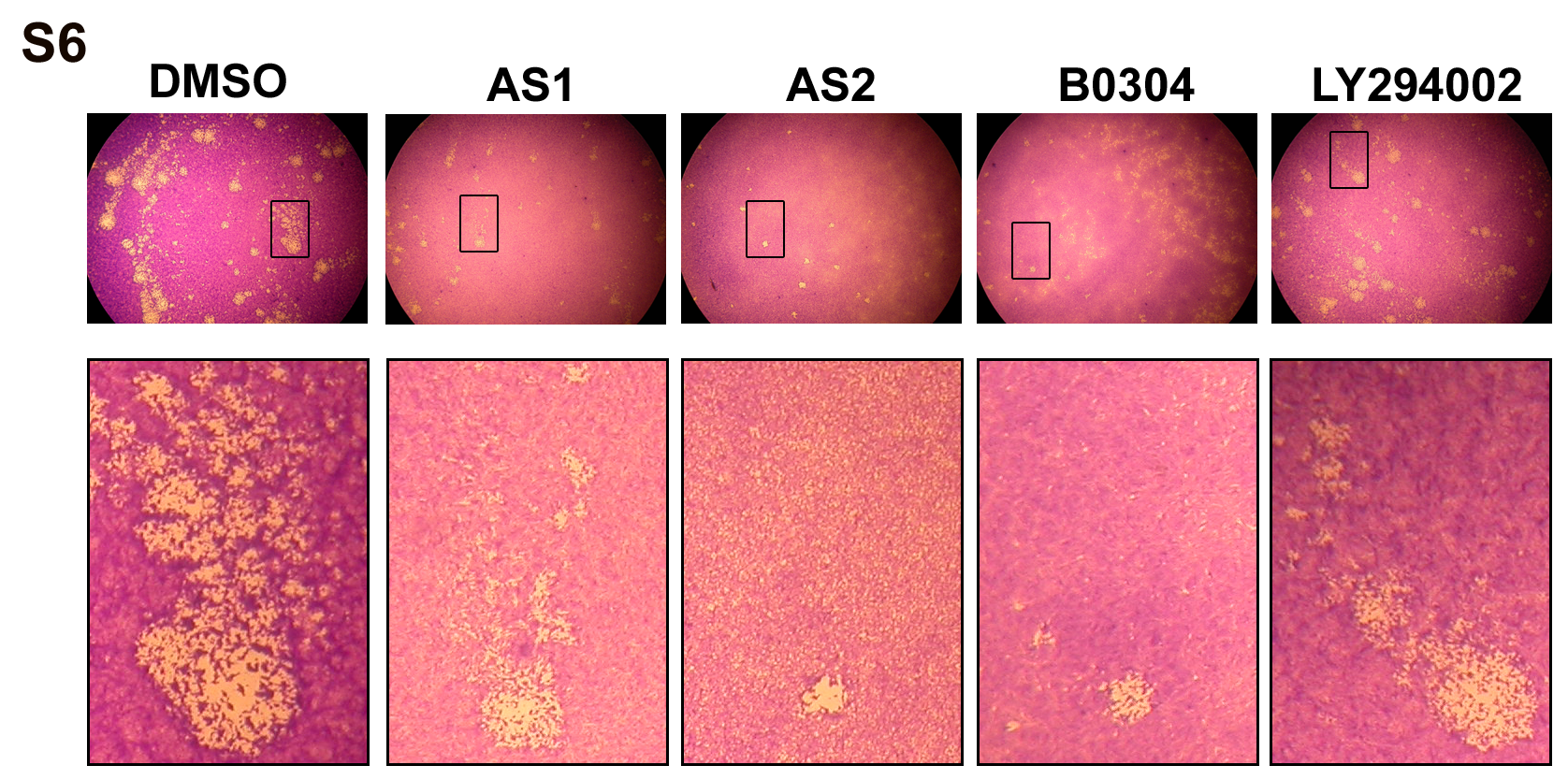

Supplement: Figure S6 — PI3K inhibitors reduce comet tails formed by VV, strain IHD-J. Insets show enlarged image of plaques boxed in top panels. Insets are at the same scale. (2.20 MB TIF) [file pone.0010884.s007.tif]

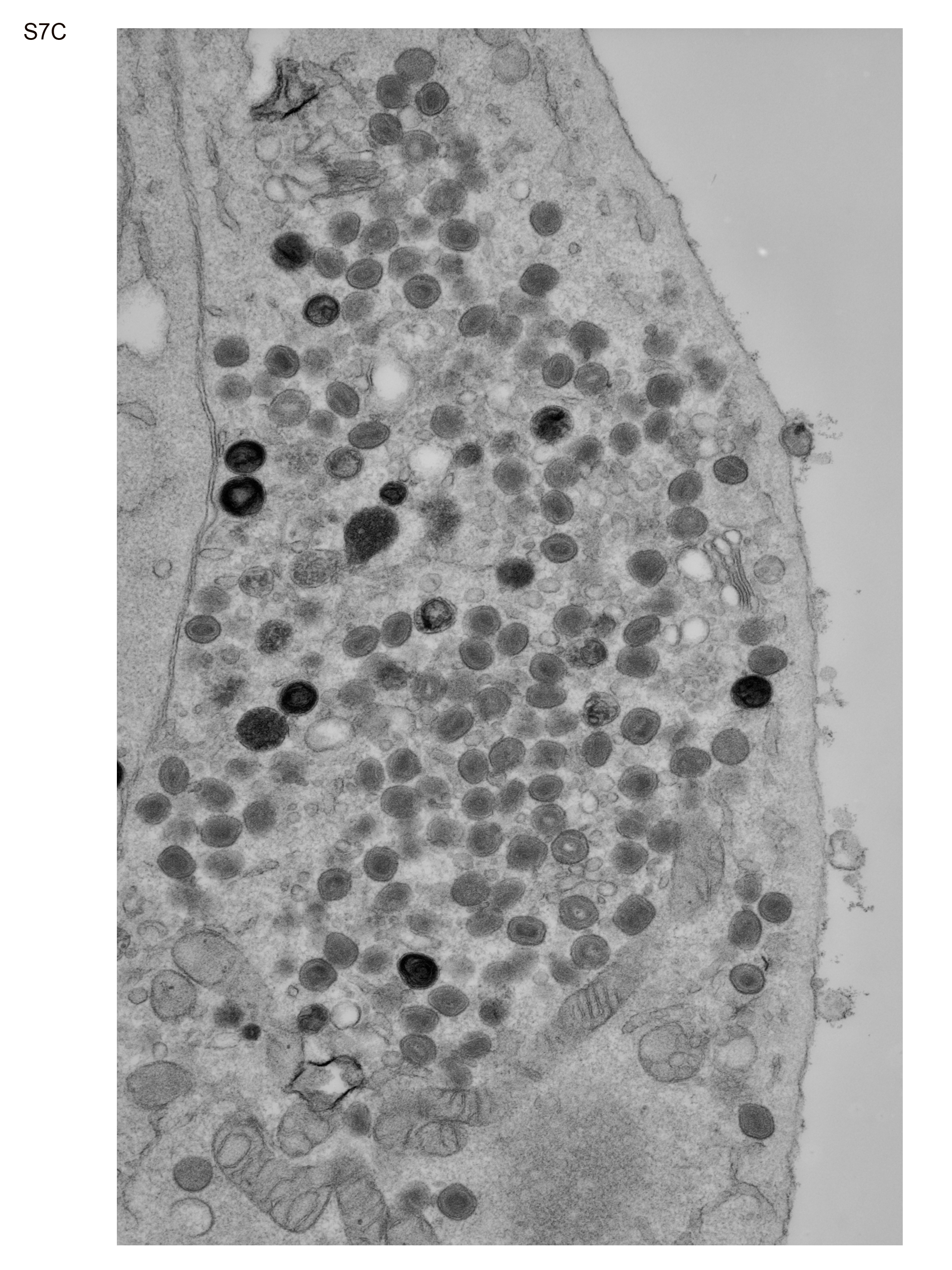

Supplement: Figure S9 — PI3K inhibitors disrupt vaccinia virus maturation. HeLa cells were infected with B5-GFP for 17 hours and treated with 50 µM AS2 (8,200X, KMnO4 fix). (5.22 MB TIF) [file pone.0010884.s010.tif]

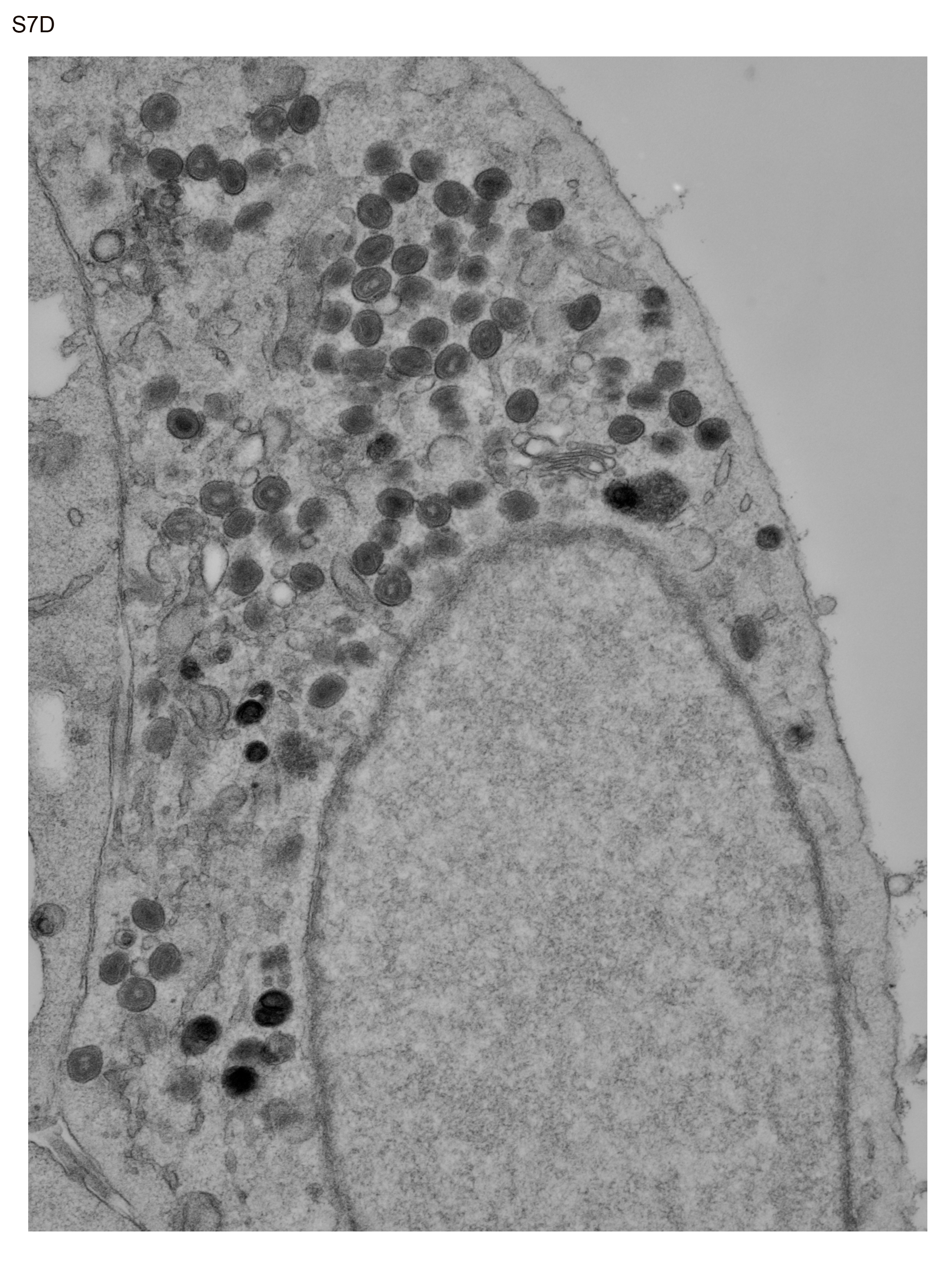

Supplement: Figure S10 — PI3K inhibitors disrupt vaccinia virus maturation. HeLa cells were infected with B5-GFP for 17 hours and treated with 50 µM AS2 (8,200X, KMnO4 fix). (5.30 MB TIF) [file pone.0010884.s011.tif]

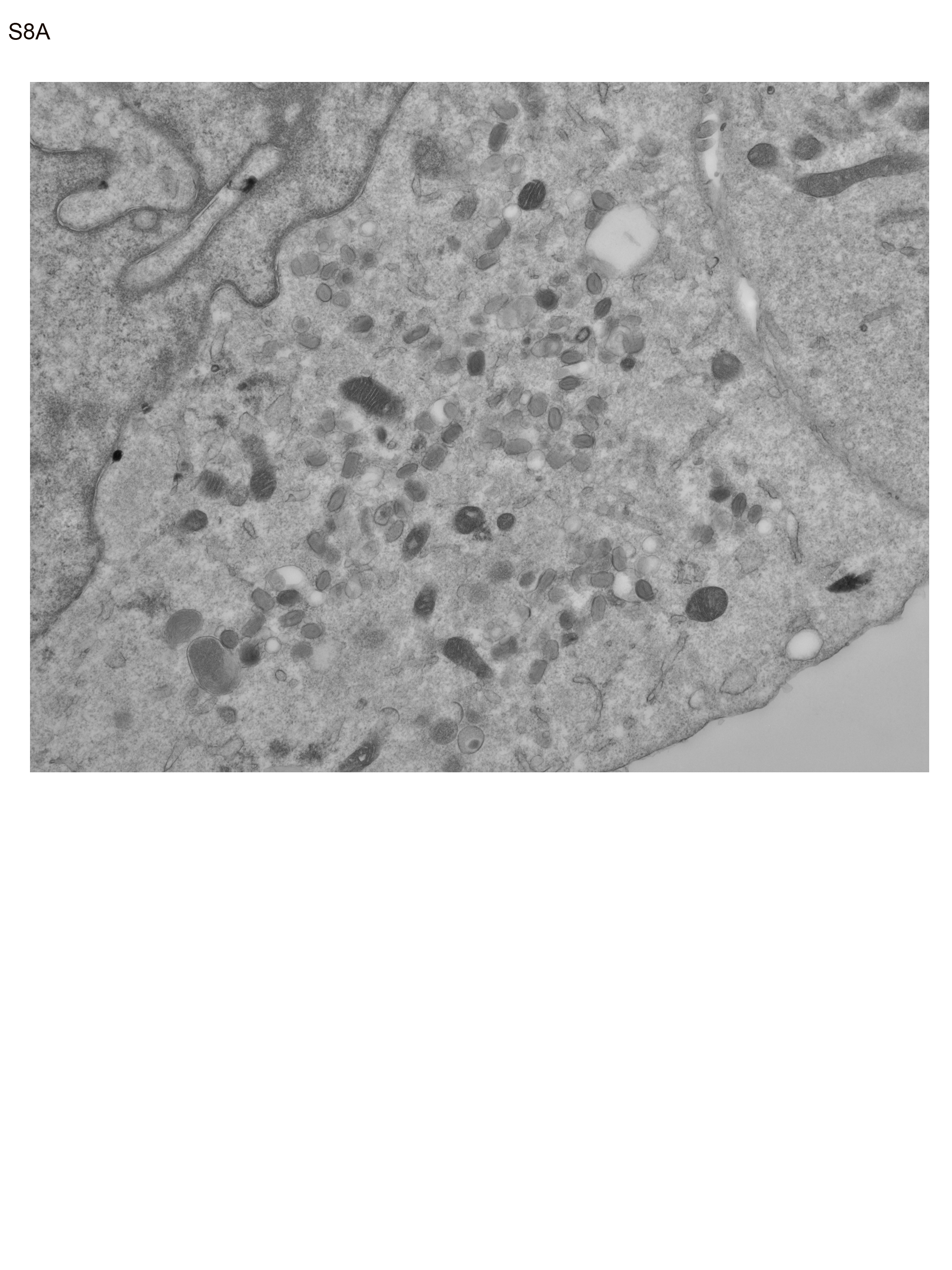

Supplement: Figure S11 — VV morphogenesis is disrupted in the p85-deficient cells. p85α−/−β−/− cells were infected with B5-GFP WR for 17 hours (2,700X, OsO4 Fix). (3.45 MB TIF) [file pone.0010884.s012.tif]

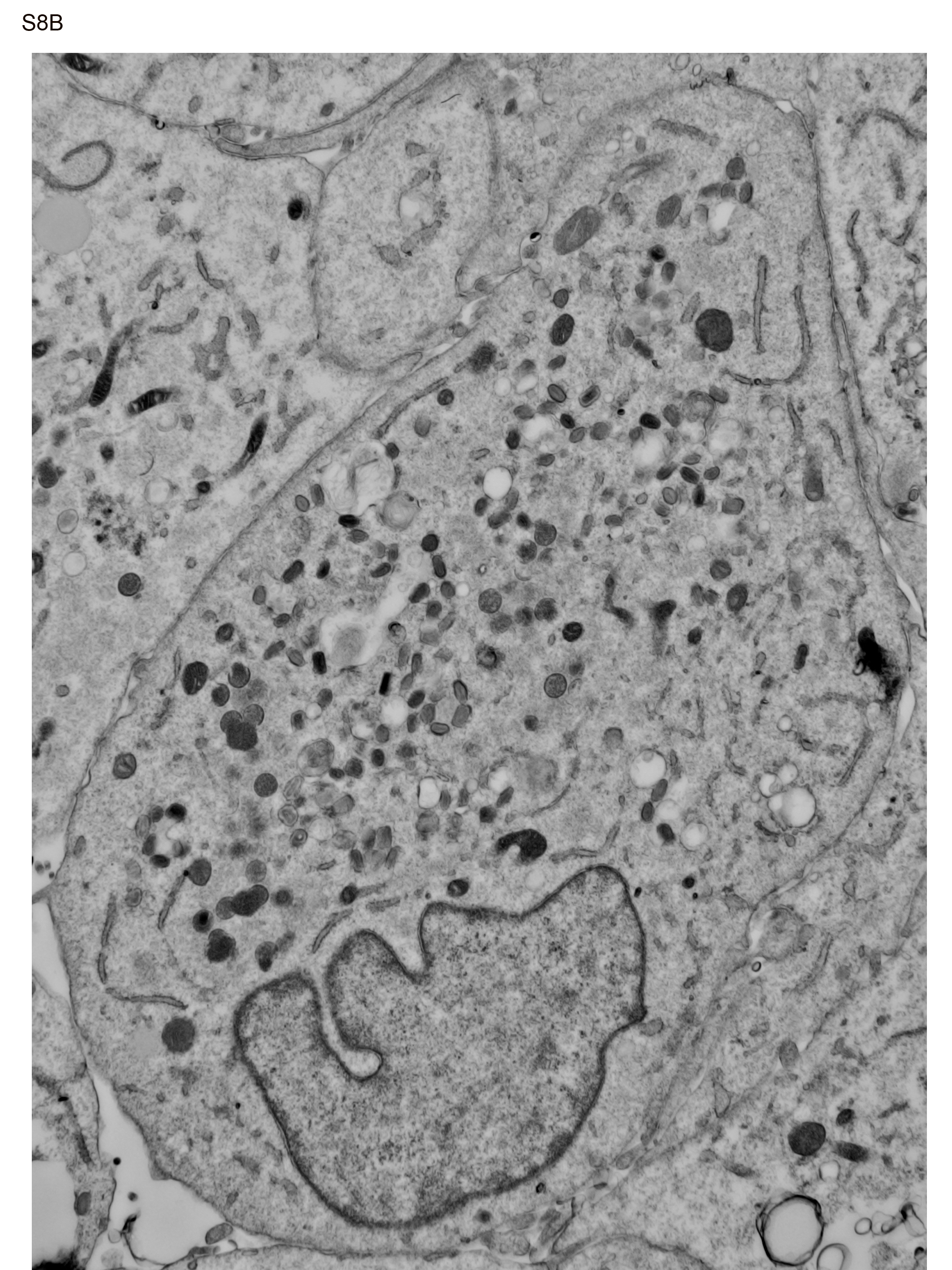

Supplement: Figure S12 — VV morphogenesis is disrupted in the p85-deficient cells. p85αα−/− β−/− cells were infected with B5-GFP WR for 17 hours (4,100X, OsO4 fix). (5.47 MB TIF) [file pone.0010884.s013.tif]

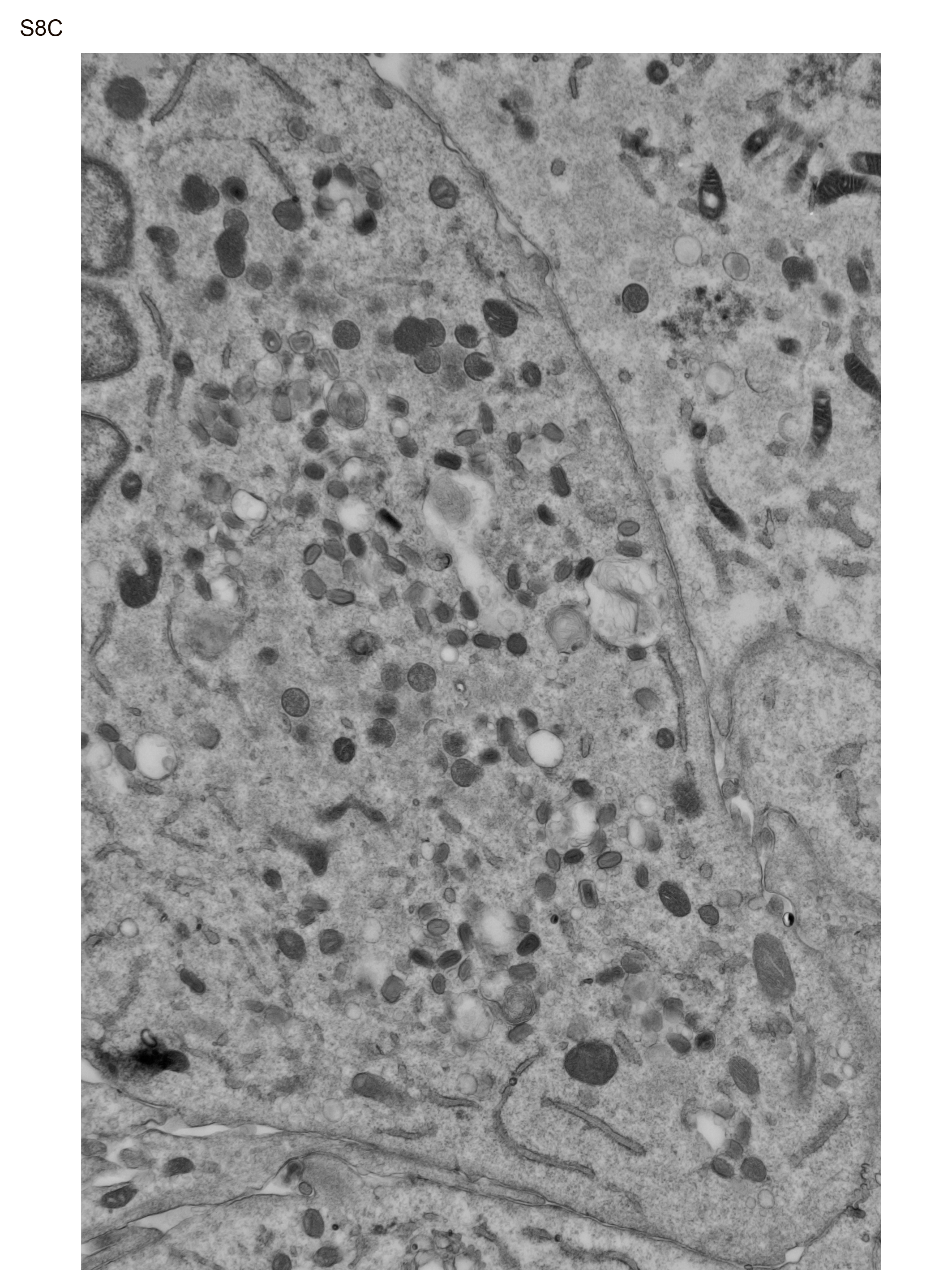

Supplement: Figure S13 — VV morphogenesis is disrupted in the p85-deficient cells. p85α−/−β−/− cells were infected with B5-GFP WR for 17 hours. (8,200X OsO4 fix). (5.07 MB TIF) [file pone.0010884.s014.tif]
